# Ion Association in Lanthanide Chloride Solutions

**DOI:** 10.1002/chem.201900945

**Published:** 2019-05-30

**Authors:** Aaron R. Finney, Sébastien Lectez, Colin L. Freeman, John H. Harding, Stephen Stackhouse

**Affiliations:** ^1^ Department of Materials Science and Engineering, Sir Robert, Hadfield Building University of Sheffield Sheffield S1 3JD UK; ^2^ School of Earth and Environment University of Leeds Leeds LS2 9JT UK

**Keywords:** ion pairing, lanthanides, molecular dynamics, potential of mean force, rare-earth elements

## Abstract

A better understanding of the solution chemistry of the lanthanide (Ln) salts in water would have wide ranging implications in materials processing, waste management, element tracing, medicine and many more fields. This is particularly true for minerals processing, given governmental concerns about lanthanide security of supply and the drive to identify environmentally sustainable processing routes. Despite much effort, even in simple systems, the mechanisms and thermodynamics of Ln^III^ association with small anions remain unclear. In the present study, molecular dynamics (MD), using a newly developed force field, provide new insights into LnCl_3_(aq) solutions. The force field accurately reproduces the structure and dynamics of Nd^3+^, Gd^3+^ and Er^3+^ in water when compared to calculations using density functional theory (DFT). Adaptive‐bias MD simulations show that the mechanisms for ion pairing change from dissociative to associative exchange depending upon cation size. Thermodynamics of association reveal that whereas ion pairing is favourable, the equilibrium distribution of species at low concentration is dominated by weakly bound solvent‐shared and solvent‐separated ion pairs, rather than contact ion pairs, reconciling a number of contrasting observations of Ln^III^–Cl association in the literature. In addition, we show that the thermodynamic stabilities of a range of inner sphere and outer sphere ${\mathrm{LnCl}}_{x}^{(3-x)+}$ coordination complexes are comparable and that the kinetics of anion binding to cations may control solution speciation distributions beyond ion pairs. The techniques adopted in this work provide a framework with which to investigate more complex solution chemistries of cations in water.

## Introduction

Lanthanides (Ln), which form the majority of the rare‐earth elements, are used extensively in technological devices due to their magnetic, electronic and optical properties.^1^ Coordination complexes of the lanthanides are particularly important in display technologies^2^ and as contrast agents in medical imaging.^3^ Furthermore, there is growing interest in lanthanide complexes as agents in the treatment of cancer and in biology.^4^, ^5^ Given that the security of supply of the lanthanides is precarious,^6^, ^7^ significant efforts are being made to identify methods to efficiently extract, process and recycle them.^8^ Separating out different lanthanides is a particular challenge that is usually performed using solvent extraction methods that tend to be expensive, hazardous and environmentally damaging.^9^ Solvent‐based processing is also used extensively in nuclear‐waste management for lanthanide and actinide separation.^10^, ^11^, ^12^ For all of the above reasons, a robust understanding of the solution chemistry of the lanthanides is of great importance.

In aqueous solutions, the lanthanides are principally found in the Ln^III^ oxidation state. Ln^III^ ionic radii decrease on moving across the series due to increasing nuclear charge, and this results in a concomitant decrease in the number of water molecules in their aquo complexes in solution, affecting their transport properties.^13^ It is widely accepted that the most likely water coordination number (CN) in the first shell for the light Ln^III^ is nine, with complexes adopting a tricapped trigonal‐prism (TTP) geometry.^14^ The CN decreases to eight for heavy Ln^III^, for which square‐antiprism (SAP) complexes are usually observed. Although earlier experiments reported water CNs for Ln^III^ in the middle of the series as eight^15^, ^16^ or nine,^17^ it is now widely accepted that the average CNs fall between these values.^18^, ^19^

Water‐exchange rates between the first and second Ln^III^ coordination spheres appear to increase across the series and reach a maximum around gadolinium before subsequently decreasing for the heavier lanthanides;^14^ however, experimental data for water exchange is incomplete for all Ln^III^. The mechanism for water exchange was proposed to be associative‐activated exchange for eight‐coordinate complexes and dissociative‐activated exchange for nine‐coordinate complexes.^14^, ^20^ Given that the relaxivity of water surrounding Gd^III^ is extremely important for its use as a contrast agent in magnetic resonance imaging, there is particular interest in the water‐exchange rates and proton relaxation for solution complexes of this lanthanide.^3^, ^21^

Exchange of the water molecules surrounding Ln^III^ with anions may occur in common electrolyte solutions to form ion pairs, trimers, etc. Of particular interest is the formation of [LnCl_*x*_(OH_2_)_*y*_]^(3−*x*)+^, not simply because Cl^−^ is a common anion in the laboratory, but also due to the high Cl^−^ concentrations, *c*(Cl^−^), that are found in many natural deposits containing elevated levels of Ln^III^.^22^, ^23^ Stability constants, *β*, provide the activities of associated species relative to those of individual constituents; for example, for Ln^III^ chloride coordination complexes, $\beta_{\mathrm{Cl}}^{x}$ (where *x* is an integer *>*0) indicate the dominant species associated with the following equilibria,

(1) $$\mathrm{Ln}{}^{3+}+x\mathrm{Cl}{}^{-}\vec{\leftarrow }\left[\mathrm{LnCl}{}_{x}\right]{}^{(3-x)+}$$

For the formation of an ion trimer (*x*=(2),

(2) $$\beta_{\mathrm{Cl}}^{2}=\frac{a({[\mathrm{LnCl}}_{2}{]}^{+})}{a\left({\mathrm{Ln}}^{3+}\right)a{\left({\mathrm{Cl}}^{-}\right)}^{2}}$$

where *a* refers to the activities of species and *β* refers to a cumulative constant which may comprise multiple stepwise‐equilibrium constants.

**Figure 1:**
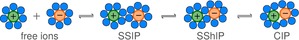
Steps in the formation of a contact ion pair (CIP) from solvated “free” ions in solution. As the cation approaches the anion, solvent molecules surrounding ions are displaced to form the solvent separated ion pair (SSIP) and the solvent‐shared ion pair (SShIP) before the CIP in a series of stepwise equilibria. Generic cations, anions and solvent molecules are shown by the green, orange and blue circles, respectively.

In the formation of the ion pair in which the ions are in direct contact (the contact ion pair (CIP)) from dissociated ions in solution, the system must first sample the solvent‐separated ion pair (SSIP) and the solvent‐shared ion pair (SShIP) states, in which one or two shared water shells separate the ions, respectively, as summarised in Figure 1. If *K* is the equilibrium constant for the stepwise formation of each type of ion pair (e.g., from the left to the right of the Scheme in Figure 1), then in theory, $\beta_{\mathrm{Cl}}^{1}=K_{\mathrm{SSIP}}\left(1+K_{\mathrm{SShIP}}\left(1+K_{\mathrm{CIP}}\right)\right)$ , but in practice, this is only true if the energetic barriers between states can be overcome on the timescales of the experiment (i.e., the system achieves a true equilibrium).

In a study by Gammons et al.,^24^ the stabilities and concentrations of NdCl_*x*_ species were determined indirectly by considering the solubility of AgCl in solutions with varying concentrations of Nd^3+^ and Cl^−^ above 40 °C. Extrapolating from the formation constants at higher temperatures, they determined that log_10_($\beta_{\mathrm{Cl}}^{1}$ ) at *T*=25 °C was 0.06±0.5 and that no further association by Cl^−^ is found at this temperature. Both $\beta_{\mathrm{Cl}}^{1}$ and $\beta_{\mathrm{Cl}}^{2}$ increased by several orders of magnitude above 50 °C. Also, by indirect measurement of solution species, Luo and Byrne^25^ found little variation in $\beta_{\mathrm{Cl}}^{1}$ across the lanthanide series. The mean log_10_($\beta_{\mathrm{Cl}}^{1}$ ) was 0.65±0.05 at *T*=25 °C by extrapolating to infinite dilution.

In line with the conclusions from earlier work by Mundy and Spedding,^26^ Allen et al.,^27^ using extended X‐ray absorption fine‐structure (EXAFS) spectroscopy, determined that outer‐sphere Ln^III^ chloride complexes are formed at low *c*(Cl^−^). When *c*(Cl^−^) exceeded about 10 mol dm^−3^, inner‐sphere complexes with average coordination 2.1 (La) to 1.1 (Eu) were observed. The decreasing coordination at constant ionic strength, *I*, suggests that the stability of chloride complexes decreases across the series. This is also consistent with the earlier data of Martell and Smith^28^ in which log_10_($\beta_{\mathrm{Cl}}^{1}$ ) was reported to be 0.48–0.23 for La–Lu (where *I*=0.1 mol dm^−3^ and *T*=25 °C). The Lawrence Livermore National Laboratory thermodynamic database^29^ provides log_10_($\beta_{\mathrm{Cl}}^{1}$ )=0.3086, log_10_($\beta_{\mathrm{Cl}}^{2}$ )=0.0308 and log_10_($\beta_{\mathrm{Cl}}^{3}$ )=−0.3203 for the association of free chlorides to Nd.

In a recent study combining X‐ray and neutron scattering and EXAFS, Díaz‐Moreno et al.^30^ found that at 1 mol kg^−1^ molalities, *b*(LaCl_3_), [LaCl(OH_2_)_8_]^2+^ is the most stable species in solution. Using Raman spectroscopy, Rudolph and Irmer^31^ found no stable lanthanum chloride complexes below 0.01 mol dm^−3^ *c*(LaCl_3_). Increasing *c*(Cl^−^) to *>*0.2 mol dm^−3^ led to [LaCl(OH_2_)_8_]^2+^ and [LaCl_2_(OH_2_)_7_]^+^. Analysis at 0.5 mol kg^−1^ using high‐energy X‐ray scattering indicated that both inner‐ and outer‐sphere erbium chloride complexes can form in solution.^32^ Using the formation constants reported by Fernández‐Ramírez et al. (log_10_($\beta_{\mathrm{Cl}}^{1}$ )=0.38, log_10_ ($\beta_{\mathrm{Cl}}^{2}$ )=0.014) and comparing different models for association, Soderholm et al.^32^ proposed that on binding, chloride adds to the waters in the first Er shell, that is, forming an inner‐sphere coordination complex.

Given the discrepancies between different experiments, it is reasonable to assume that ion pairing is controlled by kinetic factors, potentially linked to water removal around the cation. It is difficult to directly compare the relative stabilities of different LnCl_*x*_ species in experiments due to the changes in water activities associated with what are often large changes in *I*. Furthermore, the methods used to estimate formation constants are inconsistent across the experimental set. Simulations at the atomic level offer an invaluable alternative approach and have made significant advances in our understanding of Ln^3+^(aq) over the previous three decades. We note though that even here there are significant challenges associated with comparing experimental measurements, such as equilibrium constants, to calculated values from simulations. A number of computational studies of ion pairing have made progress in dealing with this challenge.^33^, ^34^, ^35^

Early classical molecular dynamics (MD) simulations by Meier et al.^36^ showed that CN in the first La^III^ coordination shell was concentration dependent (water CN and chloride CN decrease and increase, respectively, with increasing *c*(Cl^−^)), but their values of average coordination are now known to be too large. Kowall et al.^20^, ^37^ improved on this by including some polarisability in their water model which was suggested to be important to accurately capture water‐exchange dynamics. Floris and Tani^38^ developed potentials based on ab initio calculations and found the capped square antiprism (CSQA) as an alternative structure to a TTP for Ln^III^ nona‐aquo complexes. Significant understanding of Ln^3+^(aq) structure and dynamics has been provided by Duvail et al., particularly when simulations were combined with spectroscopic experiments.^18^, ^39^ They included explicit polarisability of water which was claimed to be crucial for the accurate prediction of the structure and dynamics of the first Ln coordination sphere.^40^, ^41^ Furthermore, simulations with chloride, perchlorate and nitrate allowed the authors to quantify the affinity for anion association to Ln^III^ by potential of mean force (PMF) calculations.^42^

Villa et al.^43^ developed a flexible, polarisable force field for Ln^III^ in water based on ab initio calculations. Reasonable agreement with experiments was found in the structure and dynamics of Ln^III^ aquo complexes. To achieve water‐exchange rates comparable with experiment, however, required reducing the polarisability from that calculated using ab initio measurements. Modelling explicit polarisability is thought to be necessary to accurately capture ion solvation energies.^44^, ^45^ Terrier et al.,^46^ using density functional theory (DFT), showed that the polarisation of water in the first La^III^ shell led to a +0.5 D shift in the dipole moment of water molecules compared with those in the bulk. A recent ab initio study^47^ shows that this polarisation is dominated by charge–dipole interactions for metal ions in water and that isotropic polarisability is capable of accurately modelling these systems. Migliorati et al.^48^, ^49^ have recently published pairwise intermolecular‐potential parameters for all Ln^III^ with water. Based on X‐ray spectroscopy data, with a non‐polarisable force field and rigid water, the models were able to accurately reproduce the structural properties of Ln^III^ aquo complexes. Qiao et al.^50^ also developed a non‐polarisable force field based on CHARMM and compared it to the polarisable force field of Marjolin et al.^45^ The solvation energies for the non‐polarisable model were found to be as accurate as those calculated with explicit polarisation included, suggesting that polarisability is not crucial for a good quality model.

Despite the previous work investigating Ln^III^ aqueous solutions, inconsistencies remain in our understanding of these systems, particularly with regard to cation speciation. The mechanisms by which ions associate in solution remain uncertain and the thermodynamic stabilities of coordination complexes are unclear. In the present study, MD simulations have been adopted to explore the structure and stability of [LnCl_*x*_(OH_2_)_*y*_]^(3−*x*)+^ in aqueous solutions. Nd, Gd and Er have been simulated in chloride solutions over a range of concentrations using both density functional theory (DFT‐MD) and classical empirical potential MD (C‐MD) methods. These calculations show that the effect of ion size controls both the structure and association mechanisms of coordination complexes in solution.

## Methods

We have parameterised a number of interatomic potential‐energy functions to simulate Ln^III^ in aqueous solutions. The force‐field details are provided in Table 1. Both DFT‐MD and C‐MD have been adopted in this study, with the DFT‐MD simulations and experimental data being used to benchmark the C‐MD calculations.

**Table 1:** Force‐field parameters adopted in C‐MD simulations.

Atomic species and charges			
Nd	+3*e*		
Gd	+3*e*		
Er	+3*e*		
Cl	−1*e*		
O	−0.82*e*		
H	+0.41*e*		
	
Bond potential energies: $\frac{k_{b}}{2}{\left(r-r_{0}\right)}^{2}$			
		*k*_b_ [kJ mol^−1^ Å^−2^]	*r*_0_ [Å]
O−H		4431.57	1.012
		
Bond‐angle potential energies: $\frac{k_{a}}{2}{\left(\theta -\theta_{0}\right)}^{2}$			
		*k*_a_ [kJ mol^−1^ rad^−2^]	*θ*_0_ [deg]
H‐O‐H		317.568	113.24
		
Lennard–Jones potential energies:$4\epsilon \left[{\left(\frac{\sigma }{r}\right)}^{12}-{\left(\frac{\sigma }{r}\right)}^{6}\right]$			
		*ϵ* [kJ mol^−1^]	*σ* [Å]
O O		0.65	3.165
O Cl		2.184	3.51
		
Buckingham potential energies: $A\cdot \exp\left(\frac{-r}{\rho }\right)$			
		*A* [kJ mol^−1^]	*ρ* [Å]
Nd O		58999.379	0.37
Nd Cl		193646	0.369
Gd O		58662.706	0.3551
Gd Cl		149981	0.366
Er O		58759.2	0.3477
Er Cl		143644	0.36
Cl Cl		822058.8	0.3

### System Preparation

Simulations were performed for one Ln^III^ in 5550, 555 and 111 water molecules (*b*(Ln)=0.01, 0.1 and 0.5 mol kg^−1^). The number of Cl^−^ ions was varied from zero to three depending on the particular study.

Unless otherwise stated, simulations were performed at a constant mass density and 298 K with cubic cells and 3D periodicity. The mass densities of solutions were set according to the empirical equation defined by Spedding et al.,^51^

(3) $$d=d_{0}+A_{1}m+A_{2}m^{\frac{3}{2}}+A_{3}m^{2}+A_{4}m^{\frac{5}{2}}+A_{5}m^{3}$$

In the above equation, *d* and *m* are the target mass density and concentration of the solution, whereas *d*_0_ is the mass density of water and *A_i_* are a series of density parameters fitted to experimental data that can be found in Ref. 51 and in Table S1 in the Supporting Information. We checked the validity of these densities in simulations at constant temperature and pressure.

To calculate the structural properties of Ln^III^ complexes in water, MD was performed for approximately 14–17.5 ps and 5 ns at *b*(Ln^3+^)=0.5 and 0.01 mol kg^−1^ in DFT‐MD and C‐MD, respectively. Simulations were performed with three additional Cl^−^ ions in aqueous solution at *b*(Cl^−^)=1.5 (DFT‐MD) and 0.03 mol kg^−1^ (C‐MD) as well as in pure water. Water molecules in the first Ln^III^ coordination shells were assigned using a distance criterion, in which the cutoff for Ln−O was 3.5 Å, as informed by respective time‐averaged radial‐distribution functions (RDFs). We chose to initiate C‐MD simulations with either eight (Gd and Er) or nine (Nd) water molecules in the first shell and the simulations were allowed to equilibrate over 2 ns. In the case of the DFT‐MD calculations, the first Ln^3+^ shell was set to both eight and nine water molecules in different simulations for Nd and Gd, (to ensure our sampling was not restricted by the short trajectories of DFT‐MD as the water‐exchange time is of the order of the total DFT‐MD simulation time)^14^ and eight water molecules for Er.

Dipole moments from DFT‐MD were calculated using the maximally localised Wannier function (MLWF) formalism^52^ which provides a picture of the electron distribution around atoms.^53^ This formalism was applied to configurations that were extracted every 10 fs from DFT‐MD simulations. Thus, a minimum of 100 configurations were analysed for each study of Ln^III^ complexes in water.

Umbrella Sampling (US)^54^ was applied to measure the free‐energy change for the reversible binding of Cl^−^ with Ln^3+^ (that is, Eq. (1) with *x*=1) using the Plumed plugin^55^ in C‐MD simulations at *b*(Ln^3+^)=0.01 mol kg^−1^. In order to achieve a high level of accuracy for the energy barriers between states and to gain mechanistic insight into ion association, we sampled a two‐dimensional reaction coordinate as defined by two collective variables (CVs). Harmonic‐potential energy biases were imposed to restrain the distance between Ln^3+^ and one Cl^−^ ion in the range 2.5–16 Å at either 0.25 or 0.5 Å intervals. Two additional chloride ions were restrained beyond two water shells away from Ln^3+^ (approx. 10 and 15 Å separation). In addition, the water oxygen (Ow) coordination number in the first Ln shell, as defined by the continuous function,

(4) $$S_{\mathrm{Ln}-\mathrm{Ow}}=\sum_{i}^{N}\frac{1-{\left(\frac{r_{i}-d_{0}}{r_{0}}\right)}^{n}}{1-{\left(\frac{r_{i}-d_{0}}{r_{0}}\right)}^{m}}$$

was restrained between values of 6.5 and 9.5 at intervals of approximately 0.5. In Equation (4), *N* is the number of water oxygens in the simulation and *r_i_* are the Ln−O distances. The remaining parameters are set such that the function smoothly goes from one to zero within the bounds of the first and second Ln solvation shells (for more details see the Supporting Information, Figure S1 and Table S2). Windows were added or removed depending on the system under investigation; probability distributions in the 2D reaction coordinate were monitored to ensure good overlap between CV distributions in adjacent US windows in the sampled space. See Table S3 for example US restraint parameters in the case of Gd−Cl and Figure S2 for CV probability distributions in Supporting Information. Approximately 200 US windows were simulated, each for 1 ns. Weighted histogram analysis^56^ was subsequently used to generate potential of mean force (PMF), *W*, energy maps.

Metadynamics^57^ calculations were performed to measure free‐energy differences between [LnCl_*x*_]^(3−*x*)+^ species where *x*=0–3 and *b*(Ln^3+^)=0.1 mol kg^−1^ using C‐MD. A three‐dimensional reaction coordinate was adopted, in which the three sampled CVs were *S*_Ln−Ow_ for O (of water) and *S*_Ln−Cl1_ for Cl coordination number in the first Ln shell, respectively, and also *S*_Ln−Cl2_ for Cl coordination number in the first and second Ln shells combined. Coordination of Ln by chloride was defined as in Equation (4) and the parameters for each CV can be found in the Supporting Information in Table S2. Three metadynamics calculations for each Ln system were initiated from different starting configurations, one for each of *x*=0, 1 and 2. Gaussian‐shaped bias potentials were deposited every 1000 steps with a height of 0.5 kJ mol^−1^. The width of Gaussians in the CVs for chloride coordination was 0.015, whereas for Ln–water coordination, this was 0.033. Simulations were performed for approximately 100 ns and the resulting free‐energy surfaces were generated by summing all of the deposited time‐dependent bias. Changes in free energies were then calculated as the average difference in the free energies between the same regions of CV space from three independent calculations with associated standard deviations.

### DFT‐MD computational details

Simulations were performed using the Vienna Ab initio Simulation Package (VASP),^58^, ^59^ employing the projector augmented‐wave method.^60^, ^61^ Pseudo‐potentials were generated using valence configurations of 5s^2^ 5p^6^ 5d^10^ 6s^2^ for Nd, Gd and Er (with f electrons kept frozen in the core), 3s^2^ 3p^5^ for Cl, 2s^2^ 2p^4^ for O and 1s^1^ for H. Scalar relativistic effects were accounted for, but spin‐orbit coupling was neglected. Simulations were run at *T*=341 K using the optB88^62^, ^63^ van der Waals density functional.^64^, ^65^, ^66^ Previous studies have identified this temperature as the most appropriate for DFT‐MD simulations of liquid water (the enhanced temperature compensates for over‐structuring of the water by the functional and gives a good description of the liquid water structure and self‐diffusion coefficient at ambient conditions)^67^ and is the best choice for simulations of large systems.^68^, ^69^ The kinetic‐energy cut‐off for the plane‐wave expansion was 600 eV and Brillouinzone sampling was restricted to the Γ‐point. The break condition for the electronic self‐consistent loop was set to 10^−5^ eV. Molecular dynamics simulations were performed using a time‐step of 0.5 fs, making use of the Nóse‐Hoover thermostat.^70^, ^71^ The atomic mass of hydrogen was set to 2 amu. which allows us to use a longer time step.

### C‐MD computational details

The SPC/Fw flexible three‐point water model^72^ was adopted because this has been shown to perform well in simulations of metal ions in water^73^, ^74^ as well as accurately capturing liquid‐water self‐diffusion coefficients and dielectric properties. The mass density of SPC/Fw at 298 K and 1 atm is 1012 kg m^−3^, therefore *d*_0_ in Equation (3) was set to this value. The Ln^3+^−O (water) intermolecular potential parameters were calculated by first optimising crystal structures of Nd_2_O_3_,^75^ Gd_2_O_3_ 76 and Er_2_O_3_ 77 in the GULP simulation package^78^ using the force field developed by Lewis and Catlow.^79^ Given that no Er^3+^−O^2−^ interatomic potential is included in the original force field, we generated Buckingham potential‐energy *A* and *ρ* parameters by interpolation from the parameters available for other Ln^3+^−O^2−^ interactions. The method of Schröder et al.^80^ was then used to fit the interaction potential between Ln and O. This involved scaling the Ln and O charges (*q*_Ln_=2.013 *e*, *q*_O_=−1.354 *e*), according to the charge on O in the SPC/Fw model, before optimising the Buckingham potential parameters (see Table 1) to reproduce the crystal structure with these new charges. The procedure ensures that the Pauli repulsion modelled between atoms in the solution phase is at least consistent with that for the crystalline phase.

To model the Ln–Cl intermolecular interactions, NdCl_3_, GdCl_3_ and ErCl_3_ crystal structures from experiments^81^, ^82^ were used to fit Buckingham *A* and *ρ* parameters while retaining the crystal symmetry. Crystal geometries were allowed to relax during the fitting except in the case of ErCl_3_ for which geometries were fixed because relaxation led to changes in the Er–Cl coordination environment (although the total coordination number remained constant). It is known that ErCl_3_ is unstable due to its highly hygroscopic nature, and so there is limited knowledge on the most stable crystal structure. Ln−Cl distances were monitored to ensure that the mean first coordination‐shell distances in the final structure were within 0.1 Å of the starting structure. Using this approach, it is possible to generate a spectrum of *A* and *ρ* pairs. Linear fitting of *ρ* versus *A* provides a function with which to calculate the intermolecular energies for all *A* and *ρ* parameters that conserve the first shell Ln−Cl coordination distances. We chose *A* and *ρ* based on the maximum potential energies in the short‐range Buckingham potential at the mean Ln−Cl distance following a number of tests. Although this choice does provide an upper bound to the interaction energies, changes in Buckingham energies were within thermal energy at 298 K when comparing the upper and lower bounds of the identified parameter set from crystal‐structure fitting.

Simulations were performed using the DL_POLY Classic package.^83^ Atom trajectories were obtained using a Verlet Leapfrog algorithm with a 0.5 fs timestep. A five‐chain Nosé–Hoover thermostat^84^ with a 0.1 ps relaxation time was employed to maintain the target temperature at a constant volume. US calculations were performed with one Ln^3+^, three Cl^−^ and 5550 H_2_O (i.e., *b*(Ln^3+^)=0.01 mol kg^−1^), whereas metadynamics were conducted with one Ln^3+^, three Cl^−^ and 555 H_2_O (i.e., *b*(Ln^3+^)=0.1 mol kg^−1^) and the cubic‐simulation cell parameters were set according to the target density [see Eq. (3)]. To measure solvation enthalpies, simulations containing one Ln^3+^ and 5550 H_2_O at constant temperature and pressure were performed. Here, temperature and pressure were constrained using a Nosé–Hoover thermostat and barostat with 0.1 ps and 1.0 ps relaxation times, respectively. Short‐range intermolecular interactions were truncated at a distance of 9 Å and a smooth particle mesh Ewald^85^ algorithm with 10^−7^ precision was used to calculate electrostatic potential energies. In systems with a net charge, a neutralising uniform background charge was applied using the correction of Fuchs.^86^

## Results and Discussion

### Ln^III^ aquo‐complex properties

**Figure 2:**
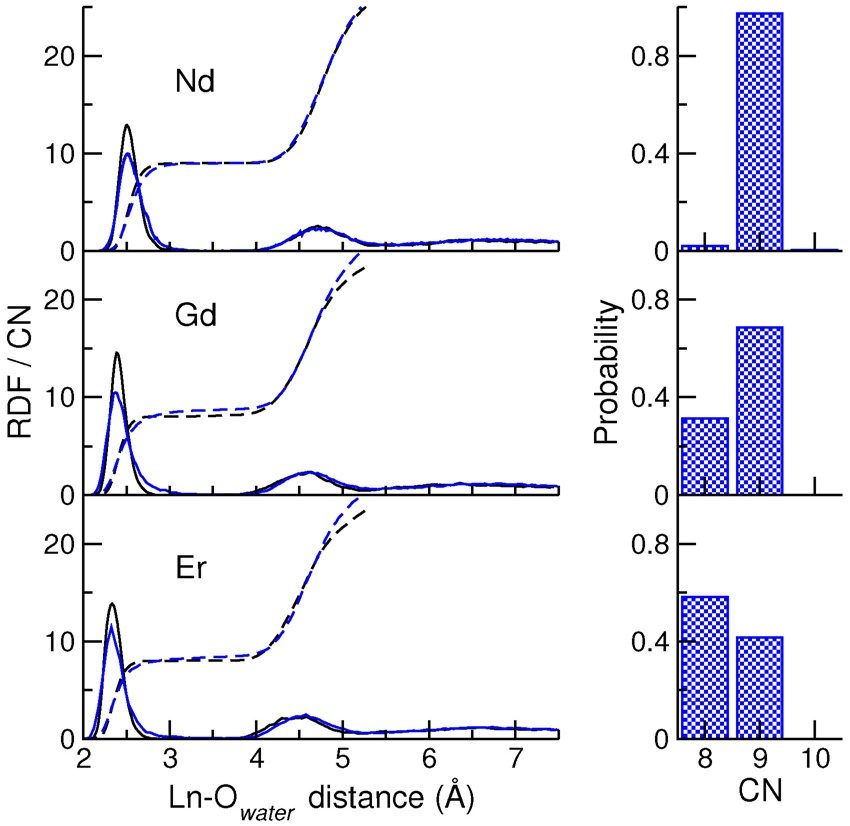
Ln^III^–O_*water*_ coordination. Left shows radial distribution functions (RDFs; solid lines) for Ln^III^–O_*water*_ where Ln is Nd, Gd or Er as indicated by labels. The integrals of RDFs (dashed lines) are also provided which indicate coordination number (CN) as a function of distance from Ln. Black lines are time averages from 12 ps DFT‐MD and blue lines are from 2.5 ns C‐MD. The probabilities for different CN values in the first shell (as defined by a 3.5 Å cutoff) are provided in the bar plots on the right.

Here we describe the results from C‐MD and DFT‐ MD simulations with Ln^3+^ and 3 Cl^−^ ions in aqueous solution. Figure 2 highlights the very good agreement in the Ln−O RDFs between the two types of simulation. The DFT‐MD RDF peaks tend to be narrower than the corresponding C‐MD ones, which can be explained by stronger Ln^III^−O interactions and/or by the shorter duration of the simulation runs. Indeed, water‐molecule exchanges between the first and second Ln^III^ coordination shells are observed less frequently in DFT‐MD calculations due to the timescale of these processes being at least on the order of the simulation times.^14^

The positions of the maxima in the first and second peaks of the Ln−O RDFs (*d*_Ln−O1_ and *d*_Ln−O2_) in Figure 2 are given in Table 2. The parameter *d*_Ln−O1_ increased by approximately 10 % on moving from the heaviest to the lightest lanthanide. The RDF peaks for second‐shell water were shifted to slightly larger distances for Gd and Er. Peak positions and coordination numbers generally compare well with other theoretical and experimental studies (see Table 2). Consistent with earlier studies^87^, ^88^ we see little evidence for a third solvent shell.

**Table 2:** The main structural, dynamical and energetic features of Ln^III^ in this work and other experimental and modelling studies. Ln–O_*water*_ distances in the first (*d*_Ln−O1_) and second (*d*_Ln−O2_) Ln coordination shells are given in units of Å. Average coordination‐number values were calculated by counting the number of water oxygens in the first (CN^1^) and second (CN^2^) hydration spheres where a distance criterion was used—informed by the positions of minima in RDFs. Water‐exchange rate constants between the first and second Ln coordination sphere, *k*_ex_, are given in ns^−1^ units. $H_{\mathrm{solv}}^{\circ }$ are in units of kJ mol^−1^ (see the discussion in the text regarding the recalculation of data from Ref. 95).

	*d*_Ln−O1_	CN^1^	*d*_Ln−O2_	CN^2^	*k*_ex_	$H_{\mathrm{solv}}^{\circ }$
Nd^III^						
DFT‐MD^[a]^	2.52	9.0	4.74	15.5		
C‐MD^[a]^	2.52	9.0	4.73	19.8	0.8–1.1	−3472
EXAFS^18^	2.53	9.0				
MD^41^	2.48	9.0	4.63	19.2	0.67	
MD^48^	2.52–2.53	8.8–8.9				
XRS^101^	2.51	8.9				
MD^43^	2.63	8.9			0.55–0.83	−3524
^17^O NMR^14^					≥0.5	
MD^44^						−3429
Expt. recal.^95^						−3501
	Gd^III^					
DFT‐MD^[a]^	2.39	8.1	4.59	14.9		
C‐MD^[a]^	2.38	8.7	4.61	17.8	3.7–5.4	−3416
EXAFS^18^	2.46	9.0				
MD^41^	2.39–2.44	8.7–9.0	4.55–4.61	18.9–19.2	2.35–3.94	
MD^48^	2.45–2.46	8.6–8.8				
^17^O NMR^14^					0.83	
MD^102^	2.44	8.6	4.65	18.1	2.69	
MD^43^	2.55	8.4			1.61–1.81	−3659
MD^44^						−3617
Expt. recal.^95^						−3601
	Er^III^					
DFT‐MD^[a]^	2.32	8.0	4.48	15.0		
C‐MD^[a]^	2.34	8.4	4.53	17.4	4.3–5.6	−3962
EXAFS^18^	2.39	8.9				
MD^41^	2.33	8.1	4.51	18.7	2.85	
MD^48^	2.39	8.0–8.3				
XRS^103^	2.37	8.2				
^17^O NMR^14^					0.13	
MD^44^						−3740
Expt. recal.^95^						−3726

[a] This work.

No inner‐sphere Ln^III^ chloride complexes formed in solution. RDFs (see the Supporting Information, Figure S3) show that in all cases, the shortest Ln–Cl distances were beyond 4 Å. When chloride was restrained to the innermost Ln^III^ coordination sphere, the cation–anion distance (Figure S1) was consistent between the DFT‐MD and C‐MD. In the second shell, Cl^−^ was most likely to be found in the hydrogen coordination sphere surrounding Ln^III^. Very little change was seen in Ln–water RDFs or CN in the second Ln^III^ shell when compared to simulations in pure water. The largest difference between the DFT‐MD and C‐MD is in the coordination distances of water hydrogen to Ln^III^ in the first shell. For example, in NdCl_3_(aq) simulations, the maximum in the first peak of Nd−H RDFs was at 3.13 and 3.24 Å in DFT‐MD and C‐MD, respectively. This is most likely due to the explicit polarisability in DFT leading to stronger water–Ln interactions and a different orientation of water molecules in the DFT‐MD compared with the C‐MD, as discussed in further detail later in the text. By the second coordination shell, RDFs for Ln−H show good agreement for both types of simulation.

Average values for the water CN in the first and second Ln^III^ coordination shells (CN^1^ and CN^2^) are provided in Table 2. CN^1^ varies linearly when modelled with the classical empirical potential. For the largest cation, there is close agreement between CN^1^ in all of the presented studies. The DFT‐MD CN^1^ for Gd appears to be too low when compared with C‐MD and other studies. Given the relatively long timescales for water exchange around Ln, DFT‐MD is unlikely to sample a representative range of equilibrium states for Ln coordination complexes in water. The value of CN^1^ for Er is not clear from the wider literature, and both the DFT‐MD and C‐MD values fall within the range reported. Figure 2 shows the probabilities for the number of water molecules in the first shell when the truncation distance for oxygen coordination was 3.5 Å. There is a 2 % probability of octa‐aquo complexes in the case of Nd and the coordination is dominated by nine water molecules. For Gd and Er, the octa‐ and nona‐aquo complexes are both observed with quite high probabilities. Larger deviations are found in both *d*_Ln−O2_ and CN^2^ in this work and the wider literature. Given that the structuring of water in Ln^III^ shells is greatly reduced beyond the first shell, it is likely that solution conditions will affect the average number of water molecules even at these distances.

**Figure 3:**
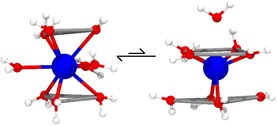
Coordination geometries adopted by [Nd(OH_2_)_9_]^3+^ in aqueous solution. On the left is the tricapped trigonal prism (TTP) which significantly dominates over the gyroelongated square antiprism (GySQAP) on the right. Nd, O and H atoms are shows as blue, red and white spheres, respectively, whereas lines highlight connections between different atoms.

Figure S4 in the Supporting Information shows O‐Ln‐O angles and Ln‐O‐*M* angle probability distributions in which *M* is the bisector between the two water O−H bonds; hence, the Ln‐O‐*M* angle quantifies the relative tilt of water molecules surrounding the cation. Bimodal distributions are observed in both DFT‐MD and C‐MD for Nd, consistent with earlier work.^41^, ^50^, ^88^, ^89^ As discussed by Qiao et al.,^50^ the peaks centred around 70° and 135° represent either the TTP or the gyroelongated square‐antiprism (GySQAP) geometries with slight deformations. The TTP geometries dominated our simulations of nona‐aquo Nd complexes but we did find a smaller population of GySQAP complexes. Representations of these structures are shown in Figure 3.

With decreasing lanthanide‐ion size, O‐Ln‐O peaks shift closer to the positions of peaks representing a SQAP geometry (75° and 142°). The shift was more pronounced in DFT‐MD simulations in the case of Gd, because the starting point here was an octa‐aquo complex with SQAP geometry. In addition to these shifts, a peak centred around 112° was found for the heavier Ln^III^ in other simulations.^41^, ^50^ This was observed in our studies as a broadening of the second peak towards smaller angles and was most notable in C‐MD simulations of Er^3+^.

The Ln‐O‐*M* angle distributions in Figure S4 (Supporting Information) show a high probability for angles close to 170° for all simulated lanthanides in C‐MD simulations. This indicates that the majority of water molecules in the first coordination sphere bind to Ln^III^ in such a way to reduce any electrostatic repulsion with positively charged water‐hydrogen atoms. The probability was reduced in the case of DFT‐MD simulations. Although the maximum probability in Ln‐O‐*M* angles was again similar for all Ln and close to 170°, peaks were much broader with angles of ∼140° showing significant probability. A similar distribution was previously calculated by Terrier et al.^46^ for La^3+^ using Car–Parrinello MD simulations. Given that the first peak in Ln–H RDFs (see Figure S3 in the Supporting Information) was at shorter distances in DFT‐MD, it is sensible to conclude that water molecules in the first shell show some hydrogen‐bonding capacity with each other as well as with water molecules in the second shell, explaining the smaller value of CN^2^ by 10–20 % in DFT‐MD than in C‐MD.

Given the above result, we compared the dipole moment of water molecules, $\mu_{H_{2}O}$ , in the first Ln^III^ coordination spheres to those of all other water molecules beyond the second shell, that is, water in bulk solution. Figure S5 (Supporting Information) provides dipole‐moment probability distributions. The dipole moment for bulk SPC/Fw water is 2.4 D with a standard deviation of 0.16 D, smaller than that measured for bulk liquid water in experiments (2.9±0.6 D)^90^ and the value estimated by Car–Parrinello MD (3.0 D).^91^ Classical liquid‐water models often underestimate experimentally determined $\mu_{H_{2}O}$ measurements.^92^, ^93^ $\mu_{H_{2}O}$ in DFT‐MD for bulk water was 3.1±0.3 D.

$\mu_{H_{2}O}$ distributions for water molecules in the first Ln^III^ coordination shells in C‐MD were shifted to greater values than those found for bulk water (5–7 % from Nd to Er). The biggest increase was for the lanthanide with the highest charge density. The distributions in the second solvation spheres were close to those for bulk water. The trend in first‐shell water perturbation was also observed in DFT‐MD simulations. Here, $\mu_{H_{2}O}$ distributions were more perturbed than for the C‐MD calculations. Indeed, in the case of Nd, Gd and Er, shifts to greater mean values by approx. 0.37, 0.54 and 0.52 D, respectively, were found to be in agreement with the results from DFT simulations of La^3+^(aq).^46^ Water‐molecule exchange between the first and second Ln^III^ coordination spheres was monitored throughout the C‐MD simulations. It was not possible to measure this with any reasonable accuracy from DFT‐MD, given the slow dynamics of the process. Any water‐oxygen atoms that were within 3.5 Å of Ln^III^ were considered to be inside the first shell. By measuring the time that water molecules reside in the first shell after first entering (*t_r_*) we generated water‐residence probability distributions, *P*(*t_r_*). Exponentials of the form, *P*(*t_r_*)=*p*(*t*_0_)exp(−*t*/*τ_r_*), were fitted to the data to estimate water mean residence times (*τ_r_*). The exchange rate (*k*_ex_) is obtained as the reciprocal of *τ_r_* and these values are provided in Table 2. Given the inherent bias associated with defining coordination according to a finite distance criterion, we also measured residence times in which the truncation distance for oxygen coordination to Ln^III^ was the maximum in the second peak of Ln−O RDFs (see Figure 2) to provide a liberal interpretation of first‐shell coordination. Only water‐molecule exchange events that persisted for at least 10 ps were considered in all our analyses.

Exchange rates for Nd^III^ compare reasonably well to those from other theoretical studies and from ^17^O nuclear magnetic resonance (NMR) experiments; however, for Gd^III^ and Er^III^, *k*_ex_ values are considerably larger than in experiments. It is worth noting the wide variation that is found in all of the calculated *k*_ex_ values in Table 2 and that MD data from other studies also predict more frequent water exchange between the first and second Ln^III^ shells than experiments. As reported in Helm and Merbach,^14^ the general consensus from ^17^O NMR studies is that *k_ex_* increases from La, reaching a maximum for lanthanides in the middle of the series before subsequently decreasing. This is explained by the transition from predominantly nine to eight‐fold coordination that is observed in the middle of the series. We observe only increasing exchange rates across the lanthanides and so it may be either that simple models cannot capture the kinetics of water exchange fully or that we are seeing a reflection of uncertainty around the experimental values. Duvail et al. found that the mean residence time for water molecules surrounding Gd decreased relative to that of Nd, but for Er this was approximately the same as Gd.^41^ The number of exchange events (water molecules either leaving or entering the first solvation sphere) per nanosecond further reflects the lack of a maximum water‐exchange rate in the middle of the lanthanide series: approximately 20, 90 and 106 ns^−1^, for Nd, Gd and Er, respectively.

The diffusion coefficients, *D*, for Nd, Gd and Er in SPC/Fw water were measured at 298 K and 1 atm at *b*=0.01 mol kg^−1^ using the Einstein relation: *D*=*MSD*/6*t* (where *MSD* is the mean squared displacement of atoms and *t* is time). *D*=4.87, 4.83 and 4.77×10^−6^ cm^2^ s^−1^ for Nd, Gd and Er, respectively, with an uncertainty of around 4 %. It is important to note that the self‐diffusion coefficient of SPC/Fw water was measured to be 2.35±0.08×10^−6^ cm^2^ s^−1^. The downward trend in *D* across the lanthanide series, though within statistical uncertainties here, and the order of *D* values are consistent with experimental and other theoretical predictions.^19^, ^44^

The enthalpies of solvation were determined as $\Delta H_{\mathrm{solv}}^{\circ }=\Delta H_{\mathrm{solution}}^{\circ }-\Delta H_{\mathrm{water}}^{\circ }+\Delta H_{\mathrm{corr}}$ from simulations at 298 K and 1 atm for *b*(Ln^3+^)=0.01 mol kg^−1^. Δ*H*_water_ was calculated from a 5 ns simulation of 5550 SPC/Fw water molecules at standard temperature and pressure. Δ*H*_corr_=Δ*H*_B_+Δ*H*_cl_+Δ*H*_comp_ provides corrections which are necessary to compare computed values to experimental data. The formulae for these corrections are provided by Kastenholz and Hünenberger in Ref. 94. Δ*H*_B_=−319.5 kJ mol^−1^ and accounts for errors in solvent polarisation from the use of finite systems with periodic boundaries. Δ*H*_C1_=−225.8 kJ mol^−1^ arises due to errors associated with the scheme used to calculate the electrostatic potential. Finally, Δ*H*_comp_=−2.3 kJ mol^−1^ is the compression work associated with solvation.

$\Delta H_{\mathrm{solv}}^{\circ }$ values from C‐MD simulations are provided in Table 2. The experimental values in Table 2 have been recalculated given that the value used in the original work by Marcus^95^ for the hydration enthalpy of a proton (−1094 kJ mol^−1^) has more recently been reassessed^96^ as −1150 kJ mol^−1^. For all cations, the simulations compare reasonably well to the experiments.^95^ $\Delta H_{\mathrm{solv}}^{\circ }$ values as predicted by Marcus for Ln^3+^ show a near linear decrease as a function of atomic number. In our work, we find that $\Delta H_{\mathrm{solv}}^{\circ }\left[{\mathrm{Gd}}^{3+}\right]$ is less negative in energy than $\Delta H_{\mathrm{solv}}^{\circ }\left[{\mathrm{Nd}}^{3+}\right]$ , but the general trend is for more negative $\Delta H_{\mathrm{solv}}^{\circ }$ across the lanthanide series. We also note that the uncertainties in our data are as high as 20 % of the mean values.

The deviations from experiment in the calculated $\Delta H_{\mathrm{solv}}^{\circ }$ here for Nd, Gd and Er are around 1, 5 and 5 %, respectively. The magnitude of the deviations are not unreasonable when compared to models which contain explicit polarisability. The force field of Martelli et al.^44^ appears to perform very well across the lanthanide series with changes *<*100 kJ mol^−1^ when compared with the values of Marcus.^95^ Thermodynamic corrections to $\Delta H_{\mathrm{solv}}^{\circ }$ were made but these are not explicitly given by the authors. $\Delta H_{\mathrm{solv}}^{\circ }$ was calculated for Nd and Gd (see Table 2) by Villa et al.^43^ Although their data appear to match well to the experimental values, they applied a +211 kJ mol^−1^ correction to $\Delta H_{\mathrm{solv}}^{\circ }$ to account for ions crossing an interface from the gas phase to the liquid phase. However, Marcus^95^ states that the absolute values provided by the experimental modelling neglect this effect to properly compare stoichiometric quantities to thermodynamic observables. When this correction is removed from $\Delta H_{\mathrm{solv}}^{\circ }$ , the deviation in the model by Villa et al. from the recalculated experimental values in Table 2 is up to 7 % (9 % when the original hydration enthalpy for a proton is used).

The C‐MD results demonstrate good agreement for our force field with experimental and other theoretical results. Given that our approach to the force‐field fitting has been systematic, and therefore is easily repeatable for other Ln cations, this procedure provides a robust and consistent tool for exploring these systems.

### Ion association

We performed two‐dimensional US calculations to understand chloride association to Ln^III^. Here, we restrained both the Ln–Cl distance and the number of water molecules in the first Ln coordination shell as defined by *S*_Ln−O_ in Equation (4). This was necessary given the slow dynamics of water exchange around the trivalent cations. Simulations were performed at *b*(LnCl_3_)=0.01 mol kg^−1^ in C‐MD. Two additional Cl^−^ were restrained beyond the second shell of Ln^3+^ to ensure that only single association by Cl^−^ was sampled. The force constants for the additional harmonic restraints were relatively small (*k*=10–20 kJ mol^−1^). Tests showed that the additional restraints did not significantly influence the relative changes in free energies between different states in the equilibrium under investigation.

**Figure 4:**
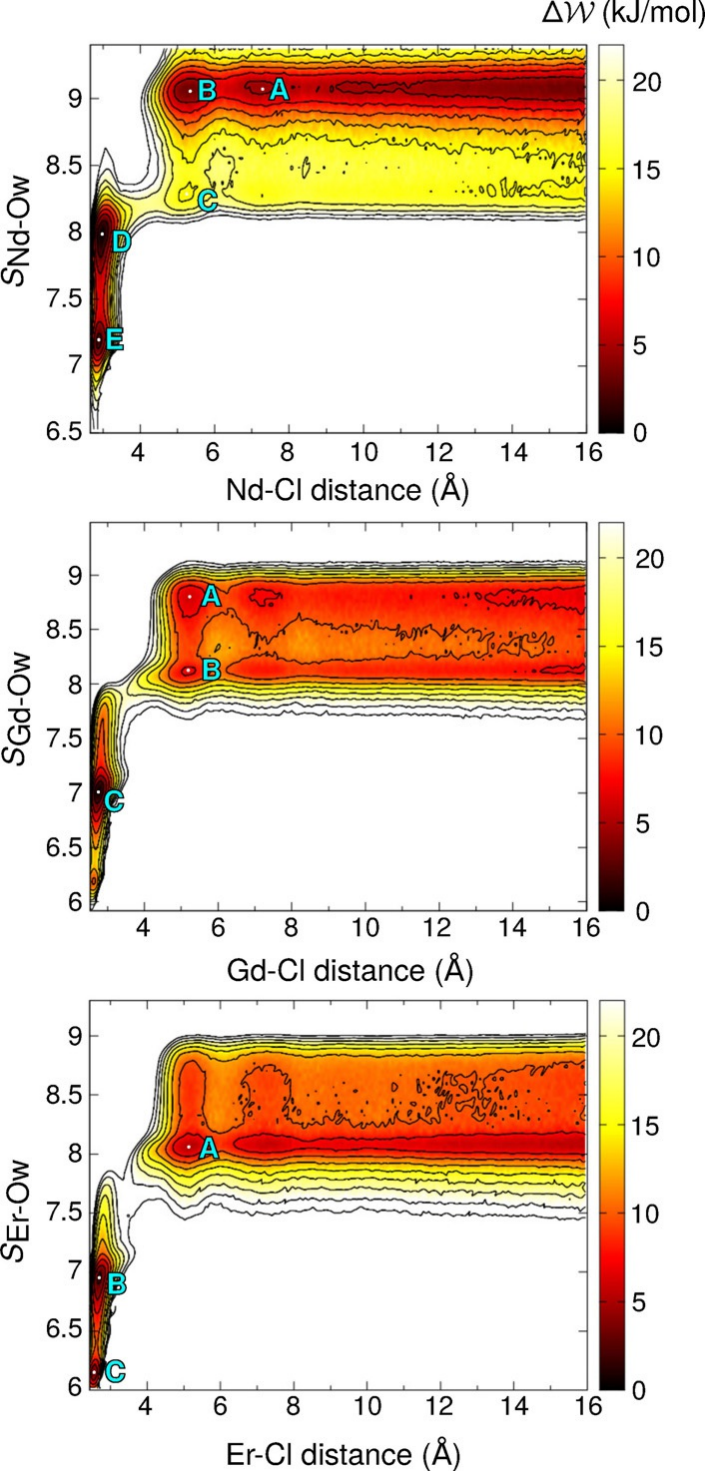
Potential of mean‐force (*W*) maps for the equilibrium given by Equation (1) (where *x*=1) for Nd, Gd and Er. The distance between Ln and Cl and the coordination of Ln with water oxygen in the first shell as a continuous function, *S*_Ln−O_, were restrained in C‐MD Umbrella Sampling simulations. Energies are given by the colour scale on the right and contour lines show changes by 1 *k*_B_ *N*_A_ *T*. Letters label features referred to in the main text.

PMFs (*W*) are provided in Figure 4 which indicate that all LnCl^2+^ CIPs were the most stable species in solution. [Nd(OH_2_)_9_]^3+^ is thermodynamically stable when Cl^−^ is beyond the second Nd^3+^ coordination sphere, in line with unbiased MD calculations. An energy barrier slightly above thermal energy, *k*_B_ *N*_A_ *T* (*k*_B_ *N*_A_ *T*≈2.478 kJ mol^−1^, where *k*_B_ is the Boltzmann constant, *N*_A_ is the Avogadro constant and *T*=298 K), is required for Cl^−^ to enter the second Nd^3+^ shell and form a SShIP, see Figure 4 point Ⓑ, from a SSIP, Ⓐ. The mean Nd–Cl distance was 5.3 Å and the TTP coordination geometry was maintained.

To produce the NdCl^2+^ CIP species, Ⓓ, a metastable minimum around 5.3 Å, ©, and approximately 4 *k*_B_ *N*_A_ *T* higher in energy than the most stable nona‐aquo complex, is first visited. The structure of the coordination complex at © resembles a distorted SQAP but the value of *S*_Ln−Ow_ is approximately 8.25, given the change in closest water molecule to cation distances. In this water‐exchange reaction, [Nd(OH_2_)_9_]^3+^⇌[Nd(OH_2_)_8_]^3+^+H_2_O, the activation energies of forward and reverse reactions were calculated to be approximately 5 *k*_B_ *N*_A_ *T* and *k*_B_ *N*_A_ *T*, respectively.

In the CIP, Ⓓ, a water molecule is lost from the Nd^3+^ inner sphere to form a more stable [NdCl(OH_2_)_8_]^2+^ in which the Nd−Cl distance was 2.91 Å. The topology of the energy surface indicates that this is a dissociative‐activated exchange pathway: one water molecule in the first coordination sphere is lost before a Cl^−^ enters. Thus, when [Nd(OH_2_)_8_]^3+^ is formed at ©, there is a competition between a Cl^−^ ion and a water molecule to move into the first Nd^3+^ shell. The corresponding activation energies were calculated as approx. 2 *k*_B_ *N*_A_ *T* and *k*_B_ *N*_A_ *T*, respectively. Given that only very limited water exchange was observed in unbiased MD simulations, this may suggest a slow rate for the forward reaction in Equation (1). The activation energy for this reaction is at least 7 *k*_B_ *N*_A_ *T*. It is possible to lose an additional water molecule to form [NdCl(OH_2_)_7_]^2+^ (Ⓔ) through an activated process (∼2.5 *k*_B_ *N*_A_ *T*), though this species is less stable by an energy at least equivalent to thermal energy. Δ*W* for Gd^III^ (shown in Figure 4) confirms the earlier findings from unbiased MD simulations. Two wide minima, Ⓐ and Ⓑ, for [Gd(OH_2_)_9_]^3+^ and [Gd(OH_2_)_8_]^3+^ are observed when Cl^−^ is beyond the first Gd coordination sphere. However, the minimum for [Gd(OH_2_)_9_]^3+^ is located at *S*_Ln−Ow_≈8.8 due to an increase in the Gd−O_*water*_ distances. At the furthest sampled distances, the higher coordination state is more stable by *<*1 kJ mol^−1^ and the energy barrier between the two states is around *k*_B_ *N*_A_ *T*. Both coordination states should, therefore, be observed in unbiased MD simulations, with [Gd(OH_2_)_9_]^3+^ being the most probable species. As Cl^−^ approaches Gd, the energy difference between the two aquo complexes grows slightly.

An energy barrier of ∼6 *k*_B_ *N*_A_ *T* separates the Gd−Cl SShIP from the more stable CIP at ©, [GdCl(OH_2_)_7_]^2+^, in which a water molecule is displaced from the inner sphere on Cl^−^ addition. The CIP has a 2.74 Å cation–anion separation distance. Given that [Gd(OH_2_)_8_]^3+^ is readily accessible when Cl^−^ is in the second coordination sphere, this marks a change in the mechanism for ion association cf. Nd^III^. Rather than losing water before chloride enters the first coordination shell in two steps, in the case of Gd, a water molecule is lost when the chloride is added to the first cation coordination sphere in a concerted step through an associative‐activated exchange mechanism. This change in mechanism can be ascribed to a decrease in cation size on moving across the lanthanide series.

For the smallest cation, Er^III^, Δ*W* shows a minimum for [ErCl(OH_2_)_8_]^3+^ when Cl^−^ is beyond the first coordination sphere. This is further confirmation for a gradual change in the most stable water‐coordination state for Ln^III^ on moving across the series. Only one minimum is found, Ⓐ, for the ErCl^2+^ SShIP. In the formation of the CIP, Ⓑ, one inner‐sphere water molecule is replaced by a chloride ion in a concerted step leaving [ErCl(OH_2_)_7_]^2+^. Similarly to Gd^III^, ion association follows associative exchange. The energy barrier to CIP formation was ∼7 *k*_B_ *N*_A_ *T* and the Er–Cl distance in the CIP was 2.65 Å. It is worth noting that the relative stability of [ErCl(OH_2_)_6_]^2+^ at © is similar to that of the most stable species at Ⓑ. That Δ*W* for [LnCl(OH_2_)_7_]^2+^→[LnCl(OH_2_)_6_]^2+^ ≈5 *k*_B_ *N*_A_ *T* and *k*_B_ *N*_A_ *T* for Gd and Er, respectively, is also indicative of the increasing cation charge density across the lanthanide series. This shifting in the stabilities of CIPs to lower coordination states may mean that the mechanism for ion association undergoes further changes as the end of the lanthanide series is approached.

A number of theoretical treatments for ion association in solution have been suggested (see the review by Marcus and Hefter, Ref. 97). In general, there is a short and a long‐range contribution to the ion‐pairing free energy. The long‐range term is governed by electrostatic forces and can be modelled by the Coulomb potential, taking the solvent as a continuum with known permittivity. Given that the permittivity of the medium attenuates the long‐range attraction between oppositely charged ions, it is crucial that this is accurately modelled in any simulation. At larger distances still, entropic forces will dominate, and dispersed ions are stabilised as infinite dilution is approached. At short ranges, theories for association differ and this is largely due to the transition from a suitable continuum‐solvent model to a discrete one and in the model for both the structure and shape of ions.

The two‐dimensional PMF surfaces (*W*(*r*, *S*)) in Figure 4 were projected onto a one‐dimensional reaction coordinate (*W*(*r*)), namely the Ln–Cl distance, by taking thermodynamic averages in *S*_Ln−O_:

(5) $$𝒲\left(r\right)=-k_{B}N_{A}T\;\ln\int_{S}\exp\left(\frac{-𝒲(r,S)}{k_{B}N_{A}T}\right)dS$$

**Figure 5:**
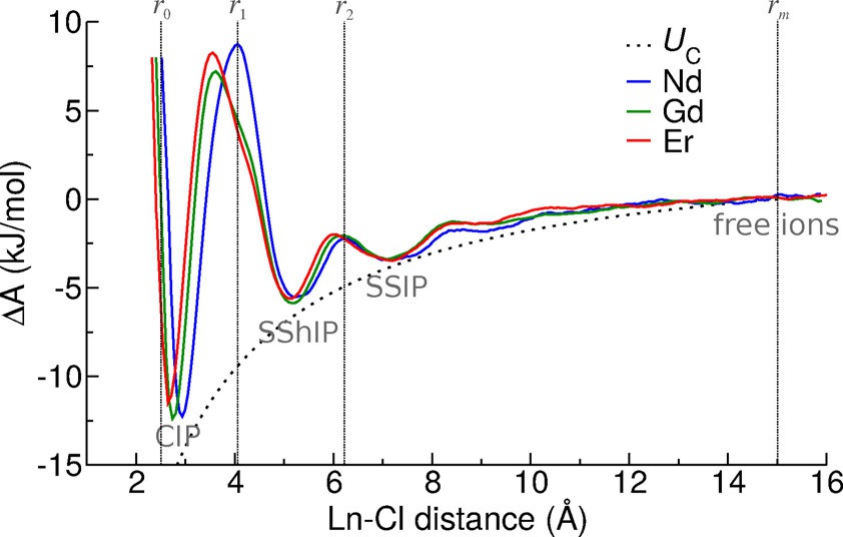
Relative free energies, Δ*A*, for the equilibrium given by Equation (1) (where *x*=1) for Nd, Gd and Er. The Coulombic particle–particle interaction energies, *U*_C_, for +3*e* and −1*e* ions in a continuum with a permittivity equal to that of bulk SPC/Fw water are also plotted. Curves were shifted such that all energies at the furthest Ln–Cl separation distances were set to zero. Vertical dashed lines show limits of integration for Nd in the calculation of equilibrium constants (see main text). Labels indicate the species associated with low energy states in different regions of Ln–Cl distance.

An entropy correction to *W*(*r*), which accounts for the increasing phase‐space volume as a function of cation–anion radial distance, allows for evaluation of changes in the Helmholtz free energies,

(6) $$\Delta A\left(r\right)=W\left(r\right)+2\;k{}_{B}N{}_{A}T\left[\ln\left(r\right)-\ln\left(r{}_{c}\right)\right]$$

where *r*_c_ is a reference state. We chose *r*_c_ to be the maximum value in the sampled Ln–Cl distances. Figure 5 shows the resulting free‐energy profiles. Calibration of the curves was performed so that the mean Δ*A* was zero in the region *r*=15–16 Å. It is important to note that the choice of calibration method can be subjective. The separation distance at which the transition between an ion pair and “free” ions dispersed in solution occurs is not well‐defined. Bjerrum showed^98^ that the minimum in the probability of finding two oppositely charged ions in solution occurs at a characteristic distance, *q*=*z*_+_ *z*_−_e^2^/4 π*ϵk*_B_ *T* (where *z* and *ϵ* are the ion charge and solution permittivity, respectively) ≈2.1 nm (with a relative permittivity of 80 for SPC/Fw water), well beyond the limit of two‐dimensional US calculations. To verify that the interaction energies between ions were suitably screened by the solvent, we calculated a free‐energy profile (by US simulation with 53×2 ns windows) as a function of just Nd–Cl separation distance to a value of *r*≈2.4 nm. From the free‐energy curve in Figure S6 (Supporting Information), we find that Δ*A* reaches a plateau around 1.4 nm. Our calibration procedure is therefore valid, and we consider ions separated by around 15 Å to be dissociated.

The effect of discrete molecular interactions during ion association is evident in the sequence of barriers that must be overcome on moving from free ions to the CIP. Minima for the SSIP, SShIP and CIP associates can be seen on decreasing Ln–Cl distance in Figure 5, separated by activation barriers which are due to solvation spheres around the cation. Only the barrier for CIP formation is substantial (up to around 15 kJ mol^−1^ for CIP formation from the SShIP); activation energies in the formation of SSIPs and SShIPs are within 2 *k*_B_ *N*_A_ *T*. Given the small energy barriers and favourable free energies of formation, a relatively large population of SSIPs and SShIPs would be expected. There is a noticeable difference in the shape of the activation barrier to forming CIPs for Nd when compared with Gd and Er which is due to the different mechanisms for ion association. The energy levels for all Ln^III^ ion pairs are very similar. Table 3 provides the change in relative free energies from free ions to CIPs. Given that there is an uncertainty of at least *k*_B_ *N*_A_ *T* in the measurement of free energy changes, it is not possible to identify a cation as forming more stable ion pairs but the trend shows more favourable binding of Cl^−^ to the lighter lanthanides which has been suggested elsewhere.^28^ The activation energies for CIP formation from SShIPs follows Nd≈Er*>*Gd, which is consistent with the idea that the frequency of water interchange between the first and second cation coordination spheres reaches a maximum in the middle of the lanthanide series.

**Table 3:** Free‐energy changes, Δ*A*, and stability constants, *K*_a_, to forming lanthanide‐chloride ion pairs from dispersed ions in solution and contact ion pairs from solvent‐shared ion pairs (*K*_CIP_; see text for details). Statistical uncertainties of around 0.2 kJ mol^−1^ apply to energy changes, but uncertainties of at least *k*_B_ *N*_A_ *T*=2.478 kJ mol^−1^ apply to the data.

	Δ*A*	log_10_(*K*_a_)	log_10_(*K*_CIP_)
Nd	−12.88	1.259	0.178
Gd	−12.77	1.198	0.092
Er	−11.91	1.188	0.024

Stability constants for ion pairing can be calculated from changes in free energies between the paired state and dispersed ions in solution. The pair correlation‐function treatment (see the discussion in the Supporting Information and Ref. 33 for derivation) has been adopted to calculate stability constants from the energy profiles in Figure 5. In the case of Ln–Cl ion pairing, the stability constant is an equilibrium constant of the form,

(7) $$K_{a}=\frac{\gamma_{ip}\;c\left({\mathrm{LnCl}}^{2+}\right)}{\gamma_{+}\;c\left({\mathrm{Ln}}^{3+}\right)\;\gamma_{-}\;c\left({\mathrm{Cl}}^{-}\right)}=\frac{\gamma_{ip}\;\alpha }{\gamma_{+}\;\gamma_{-}\;c\;{\left(1-\alpha \right)}^{2}}$$

where *γ_ip_*, *γ*_+_ and *γ*_−_ are the activity coefficients for ion pairs, cations and anions, respectively, *α* is the fraction of associated ion pairs and *c* is concentration. Given that *α* essentially represents a ratio of number densities, we can measure this through an integration of the free energy profiles in Figure 5. Furthermore—given the nature of the calculation—we can assume that the model represents ion association at infinite dilution; hence, activity coefficients are one throughout and (1−*α*)=0 within the limits of ion association.

(8) $$\lim_{\rho \to 0}K_{a}=4\;\pi \int_{r_{0}}^{r_{m}}dr\;\exp\left(\frac{-\Delta A(r)}{k_{B}N_{A}T}\right)\;r^{2}$$

In the above equation, the exponential term is the RDF and *r*_0_ and *r_m_* are the limits over which ions are considered to be associated. Here, we have used the features of the free‐energy profiles to guide the choice of integration limits. *r*_0_ was the minimum in the free‐energy curves, whereas *r_m_* was the distance at which Δ*A* deviated from zero and this was approximately 15 Å for all systems. Our approach ensures a consistent, empirical comparison between the three systems under investigation.

Because the adopted formalism is only true for systems in which ion activities equal concentrations, the resulting equilibrium constants may differ from estimates which include non‐ideal factors. We have, therefore, calculated *K_a_* values using activity coefficients based on Debye–Hückel theory which can be found in the Supporting Information. However, given the semi‐empirical nature of the theory, we focus our results here on measurements that assume nothing about the deviation from ideal behaviour that occurs in solutions at finite concentrations. This is not unreasonable, given that stability constants at infinite dilution are often quoted by extrapolating data from experiments performed over a finite concentration range.

The log_10_(*K*_a_) values are provided in Table 3. We note here that a concentration correction is made to ensure units of dm^3^ mol^−1^. Although, in theory, equilibrium constants are unitless, the constants calculated using Equation (8) do require a concentration correction in order to be consistent with Equation (7). The log_10_(*K*_a_) evaluated from simulations are consistently larger than experimentally determined log_10_($\beta_{\mathrm{Cl}}^{1}$ ) values which are usually less than one. It is important to note that this equilibrium constant accounts for the formation of associates even with many solvation shells between the cation and anion (*g*(*r*) will be above one even at 14 Å ion separation distances). The decrease in log_10_(*K*_a_) as a function of atomic number is consistent with experimental predictions,^28^ demonstrating a higher degree of ion association for the lighter lanthanides. The gradient in the decrease in log_10_(*K*_a_) as a function of atomic number here is −0.01 (*R* ^2^=0.85). The Supporting Information shows that when activity coefficients deviating from one are included in the calculation, we observe the same trend in the measured log_10_(*K*_a_) values with the same change as a function of atomic number. However, larger log_10_(*K*_a_) values are obtained using this formalism.

The free‐energy profiles in Figure 5 show that SSIP and SShIP species form spontaneously in solution with very little energetic penalty. Indeed, log_10_(*K*) for the formation of SShIPs from free ions are above one for all cations studied when the limits of integration are *r*_1_ and *r_m_* (see Figure 5). The only non‐negligible energy barrier in all of the free‐energy profiles is for the equilibrium SShIP⇌CIP. It is, therefore, informative to consider the equilibrium constant for this step (*K*_CIP_) which can be calculated according to,

(9) $$K_{\mathrm{CIP}}=\frac{\int_{r_{0}}^{r_{1}}dr\;\exp\left(\frac{-\Delta A\left(r\right)}{k_{B}N_{A}T}\right)\;r^{2}}{\int_{r_{1}}^{r_{2}}dr\;\exp\left(\frac{-\Delta A\left(r\right)}{k_{B}N_{A}T}\right)\;r^{2}}$$

where the integral limits refer to ion‐separation distances indicated in Figure 5. Table 3 gives log_10_(*K*_CIP_) data which are within the experimental range for log_10_($\beta_{\mathrm{Cl}}^{1}$ ).^28^ Again, relatively higher concentrations of CIPs should be expected for the lighter lanthanides according to these values and the change in log_10_(*K*_CIP_) with atomic number is linear (*R* ^2^=0.995) with a gradient of −0.02. These data suggest that whereas ion association is always favourable, there will be a significant population of weakly associated ion pairs in solution. log_10_(*K*_CIP_) close to zero indicates that the population distribution of species will be significantly dependent on solution conditions. Given the small driving force for CIP formation and the non‐negligible energy barrier to water removal, it is reasonable to assume that significant populations of CIPs will only be found at relatively high concentrations of free ions. If we consider the equilibrium between weakly bound states including both SShIPs and SSIPs transforming to CIPs, then log_10_(*K*) values are actually negative (−0.646, −0.708 and −0.839 for Nd^3+^, Gd^3+^ and Er^3+^, respectively). Although CIPs are energetically more stable than SSIPs and SShIPs, the conformational freedom, and therefore increased entropy, that is found for these more weakly bound states makes them collectively more probable in solution. It is likely then, that thermodynamics and kinetics both play a role in determining the concentrations of CIPs in solution.

### LnCl_3_ speciation

Three‐dimensional metadynamics calculations were performed for LnCl_3_ in water using C‐MD at 0.1 mol kg^−1^. The collective variables sampled were *S*_Ln−Cl1_, *S*_Ln−Ow_ and *S*_Ln−Cl2_, defining the coordination between Ln–Cl and Ln–OH_2_ in the first Ln^III^ coordination sphere and Ln–Cl within the first two coordination spheres, respectively (see Eq. (4) and Table 2 in the Supporting Information for details and parameters). Sampling these CVs allows us to compare the relative free energies associated with different [LnCl_*x*_]^(3−*x*)^ species and therefore to rank them in order of thermodynamic stability. In addition, the effect of anions in the second Ln coordination sphere on the stabilities of inner‐sphere complexes can be investigated.

We describe chlorides as being inner sphere, Cl^*i*^, when occupying the first coordination sphere of Ln^III^ (i.e., CIPs in the case of ion pairs) and outer sphere, Cl^*o*^, when within the second Ln^III^ coordination sphere (i.e., SShIPs in the case of ion pairs) as defined by *S*_Ln−Cl1_ and *S*_Ln−Cl2_−*S*_Ln−Cl1_, respectively. We have chosen to label species accordingly, for example, (LnCl^*i*^Cl^*o*^)^8^, which refers to a [LnCl(OH_2_)_8_]^2+^–Cl^−^ species with one chloride in the Ln^III^ inner sphere along with one solvent‐shared chloride and with a total of eight water molecules immediately surrounding the cation, as indicated by the superscript outside the brackets. Note too, that we have neglected from the labels the total charge of the species.

**Figure 6:**
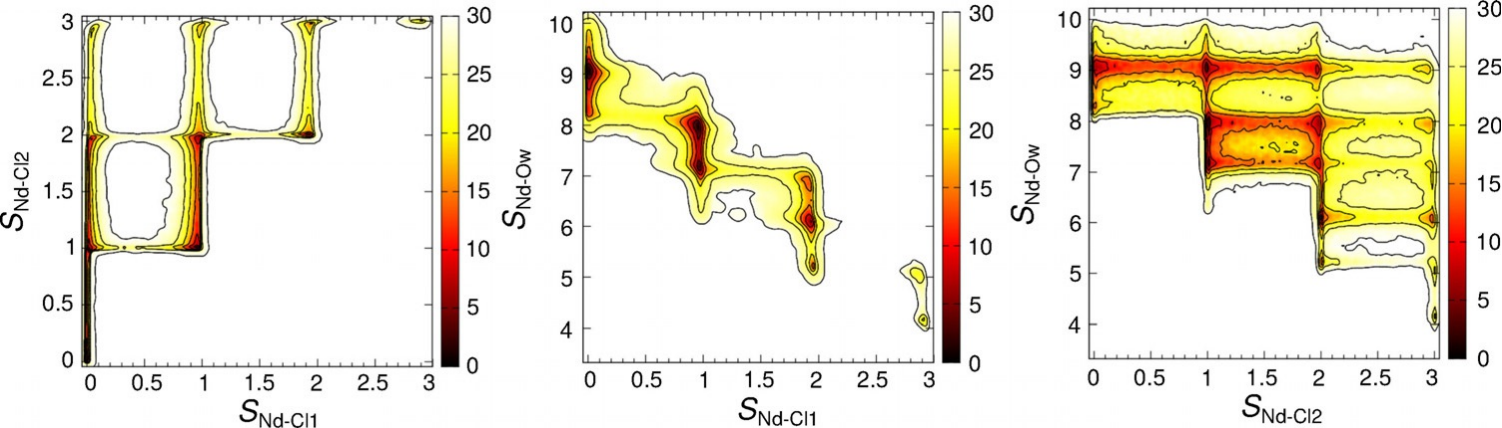
Potential of mean force energies for Nd speciation by Cl in aqueous solution. Relative energies (in kJ mol^−1^ units) are shown by the colour scale on the right. Metadynamics calculations were performed which involved the time‐dependent biased sampling of Nd with oxygen of water (*S*_Nd−O_) and chloride (*S*_Nd−Cl1_) in the first coordination sphere and chloride coordination within the first two spheres (*S*_Nd−Cl2_). Plots show projections of the four‐dimensional energy surface onto two‐dimensional reaction coordinates. Contour lines indicate energies of 2 *k*_B_ *N*_A_ *T*.

Plots representing the potential of mean‐force energy surface from a single NdCl_3_ metadynamics calculation are provided in Figure 6. Examples for GdCl_3_ and ErCl_3_ in water are provided in Figures S7 and S8 (Supporting Information). For clarity, the four‐dimensional potential of mean‐force surface was projected onto a series of two‐dimensional reaction coordinates. As a function of *S*_Ln−Cl1_ and *S*_Ln−Ow_, we find a minimum for a nona‐aquo complex. An energy barrier must be crossed before chloride addition leads to (NdCl^*i*^)^8^, which is the most stable species in solution, as indicated by the relative energies for some of the sampled species listed in Table 4. It should be noted that whereas Table 4 provides the statistical uncertainty from averaging multiple metadynamics calculations, there is an uncertainty of at least *k*_B_ *N*_A_ *T* in all of the energy changes reported.

**Table 4:** Relative potential of mean‐force energies of ${\left({\mathrm{LnCl}}_{x_{1}}^{i}{\mathrm{Cl}}_{x_{2}}^{o}\right)}^{y}$ at 0.1 mol kg^−1^. In species labels, Cl^*i*^ and Cl^*o*^ refer to inner and outer sphere chloride, respectively, whereas the superscript *y* outside of brackets indicates the number of water molecules immediately surrounding the cation. Energies are in units of *k*_B_ *N*_A_ *T* (2.478 kJ mol^−1^ at *T*=298 K). Statistical uncertainties give one standard deviation in the relative energies from multiple metadynamics calculations.

(NdCl^*i*^)^8^	0±0.44		(GdCli2 )^5^	0±0		${\left({\mathrm{ErCl}}_{2}^{i}\right)}^{5}$	0±0.54
(NdCl^*o*^)^9^	0.2±0.64		(GdCl^*i*^)^7^	1.63±0.6		${\left({\mathrm{ErCl}}_{3}^{i}\right)}^{3}$	0.96±1.43
(Nd)^9^	0.25±0.6		(GdCl^*o*^)^9^	2.26±0.97		(ErCl^*o*^)^8^	4.57±1.27
(NdCl^*i*^)^7^	1.12±0.44		(Gd)^9^	2.55±1.17		(ErCl^*i*^)^7^	4.61±0.61
${\left({\mathrm{NdCl}}_{2}^{i}\right)}^{6}$	1.7±0.86		(GdCl^*i*^Cl^*o*^)^7^	3.24±0.88		(Er)^8^	4.79±0.73
(NdCl^*i*^Cl^*o*^)^8^	2.13±0.35		${\left({\mathrm{GdCl}}_{2}^{i}{\mathrm{Cl}}^{o}\right)}^{5}$	4.42±0.30		${\left({\mathrm{ErCl}}_{2}^{i}{\mathrm{Cl}}^{o}\right)}^{5}$	5.39±0.92
${\left({\mathrm{NdCl}}_{2}^{o}\right)}^{9}$	2.78±0.42		${\left({\mathrm{GdCl}}_{3}^{i}\right)}^{4}$	5.31±0.80		(ErCl^*i*^Cl^*o*^)^7^	6.36±0.91
${\left({\mathrm{NdCl}}_{3}^{o}\right)}^{9}$	7.07±0.44		${\left({\mathrm{GdCl}}_{2}^{o}\right)}^{9}$	5.43±0.48		${\left({\mathrm{ErCl}}_{2}^{o}\right)}^{8}$	7.78±1.04
${\left({\mathrm{NdCl}}_{3}^{i}\right)}^{5}$	7.57±0.84		${\left({\mathrm{GdCl}}_{3}^{o}\right)}^{9}$	9.39±0.67		${\left({\mathrm{ErCl}}_{3}^{o}\right)}^{8}$	12.32±0.94

An outer‐sphere ion pair, (NdCl^*o*^)^9^, is slightly higher in energy than the inner‐sphere ion pair and both are more stable than the lanthanide immediately surrounded by water. All of this is consistent with the results of our US calculations (see Figure 5). The energy differences between these species are smaller than those found in US calculations at *b*(Ln) = 0.01 mol kg^−1^, highlighting the effect of concentration on speciation (also note that entropic corrections for Nd–Cl radial distance are not included here). An inner‐sphere complex containing two chlorides, ${\left({\mathrm{NdCl}}_{2}^{i}\right)}^{6}$ , was less stable than Nd immediately surrounded by nine water molecules by about *k*_B_ *N*_A_ *T*. The energy barrier to this state from (NdCl^*i*^)^7^ was >10 *k*_B_ *N*_A_ *T*. Gammons et al.^24^ did not detect any [NdCl_2_]^+^ in aqueous solutions below 100 °C. Although free Nd and [NdCl]^2+^ are more stable, any thermodynamic analysis for systems at elevated concentrations (such as in hydrothermal fluids or brines) should predict a relatively small population of ${\left({\mathrm{NdCl}}_{2}^{i}\right)}^{6}$ based on the calculations presented here. We analysed the structure of ${\left({\mathrm{NdCl}}_{2}^{i}\right)}^{6}$ and found a distorted SQAP geometry with chlorides positioned to maximise the dipole moment (see Figure S9 in the Supporting Information). In their review, Migdisov et al.^23^ discuss the geometries of LnCl_2_^+^. It is suggested that this structure which was found for LaCl_2_^+^ by Petit et al.,^99^ could result from a favourable chloride coordination geometry that differs from that of water. This arrangement does allow for water molecules in the second shell to bind effectively to both water and chloride in the first shell.

A number of metastable minima were found in the energy landscape which contained two associated chlorides either as a combination of inner and outer‐sphere, (NdCl^*i*^Cl^*o*^)^8^, or as both outer‐sphere, ${\left({\mathrm{NdCl}}_{2}^{o}\right)}^{9}$ , ions. Five minima are shown in Figure 6, with a reaction coordinate defined by *S*_Ln−Cl2_ and *S*_Ln−Ow_, due to a range of inner and outer‐sphere coordination states and a varying number of water molecules in the first Nd coordination spheres. These were all ≳2 *k*_B_ *N*_A_ *T* higher in energy than the most stable species. (NdCl^*i*^Cl^*o*^)^8^ was relatively accessible, being separated from (NdCl^*o*^)^8^ by an energy barrier of around 5 *k*_B_ *N*_A_ *T*, indicating that the thermodynamic barrier to the formation of a CIP is lowered when Cl^−^ is in the second coordination sphere of the cation. This is an important observation because reduced thermodynamic energy barriers should result in an increase in the rate at which cation binding to other ligands takes place, assuming the mechanisms for association are the same. The energy barrier to move one chloride from the inner to outer sphere of (NdCl^*i*^Cl^*o*^)^8^ to form ${\left({\mathrm{NdCl}}_{2}^{o}\right)}^{9}$ is around 10 *k*_B_ *N*_A_ *T*.

All of the coordination states in which three chlorides were bound to Nd, either in the inner or outer coordination sphere, were relatively high in energy. Energy barriers to adding chloride into coordination complexes which already contained two chlorides were not particularly large; however, the disturbance of water structure when three chlorides surround Nd is clearly unfavourable. The relative energy of ${\left({\mathrm{NdCl}}_{3}^{i}\right)}^{4}$ compared with the most stable complex was around +20 kJ mol^−1^. It is very unlikely, therefore, that this species would be observed under standard conditions.

The speciation of Gd in chloride solutions under the examined conditions was different to that for Nd. Greater chloride coordination to the cation was thermodynamically favourable, with ${\left({\mathrm{GdCl}}_{2}^{i}\right)}^{5}$ being the most stable species in all metadynamics calculations. The complex has a pentagonal‐bipyramidal geometry (see the Supporting Information, Figure S9). This structure is consistent with those expected from valence shell electron pair repulsion (VSEPR) theory. The energy barrier to forming ${\left({\mathrm{GdCl}}_{2}^{i}\right)}^{5}$ from a CIP was, however, above 13 *k*_B_ *N*_A_ *T*, and given that there is a non‐negligible activation energy to ion pair formation, the concentration of these species is likely to be extremely low despite their stability. Furthermore, the energy difference between this species and ion pairs is within the range of thermal fluctuations, and so there is not a significant driving force for the addition of another anion. As was found for Nd, inner‐sphere ion pairs, outer‐sphere ion pairs and the solvated Gd‐cation energies follow the same order predicted in our US calculations, followed by ion‐trimer associates with both inner and outer sphere chlorides. Ion quartets with chlorides in the first and second shell were at least 5 *k*_B_ *N*_A_ *T* less stable than the most stable species and given that the energy barriers to forming these associates was above 12 *k*_B_ *N*_A_ *T*, they are unlikely to be detected at 298 K.

The smallest cation (Er) showed the most stable high‐coordination states. Table 4 shows that Er bound to two and three chlorides in the first coordination sphere were the most stable species. Figure S9, Supporting Information shows that ${\left({\mathrm{ErCl}}_{3}^{i}\right)}^{3}$ has a distorted octahedral geometry. As in the case of ${\left({\mathrm{GdCl}}_{2}^{i}\right)}^{5}$ , the geometry of ${\left({\mathrm{ErCl}}_{3}^{i}\right)}^{3}$ is what can be expected from VSEPR theory. Again, the activation energies to forming these high‐coordination species was considerable: adding a chloride to the inner sphere of ${\left({\mathrm{ErCl}}_{2}^{i}\right)}^{5}$ required *>*40 kJ mol^−1^. Following these species, inner and outer‐sphere ion pairs and the solvated cation were equally stable within uncertainties, providing further indication that concentration changes can shift equilibria. Associates containing multiple chlorides in a combination of inner and outer sphere geometries were relatively high in energy, though when compared to the energies of CIPs, the energy differences were approximately the same as for Gd and Nd. Outer‐sphere ion trimers and quartets were unstable by up to around 30 kJ mol^−1^.

The data in Table 4 may appear to suggest that high levels of cation–anion coordination should be expected in LnCl_3_ aqueous solutions. As stated above, however, there are considerable activation energies to forming these species and so concentrations are likely to be very low; nonetheless, the data do show that high‐coordination states are either of similar stability to or greater stability than free ions and ion pairs (under the chosen conditions). This is not unreasonable considering the enthalpic gain in coupling positive and negative ions, as well as the entropic gain associated with releasing tightly bound water molecules from the cation coordination sphere. It appears that as cation size decreases, the relative stabilities of ion trimers and quartets increases, and this could be due to the increasing charge density of the lanthanides across the series. In general though, the order of stabilities from ion pairs to larger associates is consistent for all three cations. Although it may appear that the range of relative stabilities for different associates increases from Nd to Gd and Er, the energy differences for for example, adding a chloride to the outer sphere of the most stable CIP is ∼2 *k*_B_ *N*_A_ *T* for all cations. Current thermodynamic‐modelling packages rarely consider species beyond free ions, CIPs and inner‐sphere‐coordinated ion trimers. For most applications this is probably adequate, particularly because association constants in these codes can capture a distribution of coordination complex types into a single value. However, for a detailed analysis of the chemistries of the lanthanides in solution, our results suggest that consideration of species beyond simple pairs and trimers is important. This is especially the case in hydrothermal fluids in which increasing temperatures should reduce the barriers to anion binding.

## Conclusions

The force field developed in this work performs well in predicting the behaviour of the lanthanides in water. This was confirmed by calculations at the DFT level and by comparison to experimental data. Furthermore, the enthalpies of solvation highlight that the force field can accurately model the thermodynamics of cations in water. The largest deviations in the comparison between calculated and experimental data was for erbium. This is not surprising, given that the force‐field fitting procedure relies upon well characterised, stable crystal structures. This computationally inexpensive force field, nonetheless, performs well enough to understand lanthanides in solution. Our method is also highly systematic, allowing for the addition of other Ln cations and impurities/additives.

Two‐dimensional US calculations depict clearly that there is a transition in the relative stabilities of nine versus eight‐coordinated Ln^III^ aquo complexes, as has been shown in other studies.^19^ Given that the relative free energies for [Gd(OH_2_)_9_]^3+^ and [Gd(OH_2_)_8_]^3+^ are approximately equal, it is likely that the transition occurs at or close to this cation in the lanthanide series. The water‐coordination number linearly correlates with the ionic radii of the lanthanides investigated (CN=−0.075*Z*+13.5, where *Z* is atomic number; *R* ^2^=1). These factors also control the mechanism for ligand exchange. A clear shift from dissociated exchange (Nd), akin to an elimination‐style process in which the first step is water removal from the inner Ln^III^ sphere, to associated exchange (Gd and Er), in which water is lost and chloride is added to the inner sphere in a concerted step, was apparent from potential of mean‐force maps when both Ln–Cl distance and Ln–water coordination were examined. This is consistent with a change in the mechanism of water interchange, between the first two cation‐solvation spheres, around the middle of the series as proposed elsewhere.^15^, ^19^

The free‐energy profiles in Figure 5 show that, for all of the lanthanides examined, CIPs are the most thermodynamically stable species in the equilibrium in Equation (1) when *x*=1. From dispersed ions in solution, the formation of weakly bound SSIPs and SShIPs is both favourable and incurs little energetic cost with thermodynamic barriers within ∼2 *k*_B_ *N*_A_ *T*. The removal of water to form CIPs from SShIP states incurs an energetic cost of around 15 kJ mol^−1^ for all cations. This barrier is very unlikely to be surmounted on the timescales of the equilibrium simulations. Furthermore, CIPs may be inaccessible in some experiments, but this energy barrier is certainly accessible on geological timescales and in experiments that are allowed to establish a true equilibrium. Duvail et al.^42^ performed one‐dimensional PMF calculations for the binding of Cl to Nd^III^. They found a barrier to forming a CIP from a SShIP of 15 *k*_B_ *N*_A_ *T* which is larger than our estimate of 8 *k*_B_ *N*_A_ *T*. In addition, the SShIP showed approximately the same energy as the CIP. However, no entropy correction was added to the energy profiles to properly consider thermodynamic activation barriers and species stabilities. We believe that if these were included then the Duvail et al. free energies would show the same ordering of thermodynamic stabilities for ion pairs that we present and smaller activation energies to forming ion pairs.

By considering different types of ion pairs on the pathway from free ions to CIPs, our analysis shows that whereas ion association is always favourable, the equilibrium constant for the formation of CIPs from SShIPs (*K*_CIP_) is close to one for all cations within thermal fluctuations (with a trend to smaller values across the series). The energy change to forming CIPs, calculated using Δ*A*=−*k*_B_ *N*_A_ *T*ln(*K*), is within the range −0.14 to −1 kJ mol^−1^. This means that the presence of CIPs will be very dependent upon the solution conditions. It is interesting to note that our estimate for this equilibrium constant is close to the experimentally determined values but that our measurements of log_10_(*K*_a_), which account for the formation of all ion associates, is much larger than the reported values of log_10_($\beta_{\mathrm{Cl}}^{1}$ ), even when activity coefficients deviating from unity are considered (see the Supporting Information). Nonetheless, the trend in decreasing log_10_(*K*_a_) across the lanthanide series was evident. Our analysis also shows that the concentrations of contact pairs should be much lower than the more weakly bound ion pairs (i.e., SShIPS, SSIPs and beyond) if one can consider a single equilibrium between these types of ion associates: log_10_(*K*) was below zero for all cations and there was a trend to more negative values for the heavier lanthanides.

Previous experimental and theoretical studies have failed to provide consistent conclusions about the nature of chloride association with lanthanides in solution. Stability constants have been calculated^24^, ^25^, ^28^ and, whereas these are widely varying, they suggest that ion pairs are stable with respect to dispersed ions. In contrast, spectroscopic measurements and theoretical studies^27^, ^31^, ^32^, ^48^, ^100^ suggest that chloride preferentially forms outer‐sphere complexes with lanthanides—only at high salt concentrations are contact ion pairs formed. Our study unites these depictions of ion pairing. Ion association is always favourable, which is not surprising given the strong Coulombic attraction between +3*e* and −1*e* ions in water, but weakly bound ion pairs are likely to dominate the equilibrium distribution under relatively mild conditions both thermodynamically and because of the relatively large energy barrier for the removal of water in the first coordination sphere of Ln^III^. At higher concentrations, disturbance in the structure of water‐surrounding cations is likely to lead to a decrease in the thermodynamic energy barriers and the equilibrium distribution of ion pairs increases simply because water liberation becomes more favourable. Crucially then, contact‐ion‐pair formation is controlled both by kinetic and thermodynamic factors.

Calculations which sampled multiple [LnCl_*x*_(OH_2_)_*y*_]^(3−*x*)^ species highlighted the wide number of possible ion associates that can form in solution. Often in speciation analysis, one or two equilibria are considered for the association of chloride to cations; however, our data show that there is a multitude of equilibria associated with the formation of both inner and outer‐sphere complexes. Although multiple equilibria can be averaged into just one stability constant, it is important to recognise that estimation of species concentrations from a single stability constant is not straightforward. Highly coordinated states become more favourable towards the end of the lanthanide series; however, the activation energies to forming these species make them inaccessible at 298 K. In geological settings, such as hydrothermal deposits, it is likely that there is a wide range of possible association states that could be considered beyond contact ion pairs and trimers.

## Conflict of interest

The authors declare no conflict of interest.

## Supporting information

As a service to our authors and readers, this journal provides supporting information supplied by the authors. Such materials are peer reviewed and may be re‐organized for online delivery, but are not copy‐edited or typeset. Technical support issues arising from supporting information (other than missing files) should be addressed to the authors.

Supplementary
