# Supplementary material for: Ion Association in Lanthanide Chloride Solutions
Source: Chemistry. 2019 May 30;25(37):8725–40. doi: 10.1002/chem.201900945 (PMC6619345; doi:10.1002/chem.201900945)
Supplement: Supplementary file 1 — Supplementary [file CHEM-25-8725-s001.pdf]

CHEMISTRY
A European Journal

Supporting Information

Ion Association in Lanthanide Chloride Solutions

Aaron R. Finney,*[a] Sébastien Lectez,[b] Colin L. Freeman,[a] John H. Harding,[a] and Stephen Stackhouse*[b]

chem_201900945_sm_miscellaneous_information.pdf

Table S1 *Parameters taken from Spedding et al.* [1] *for target solution mass densities (see Equation 3 in main article).*

	A_1	A_2	A_3	A_4	A_5
$NdCl_3$	0.238275	-0.012536	0.001171	-0.006628	0.001440
$GdCl_3$	0.246545	-0.004738	-0.011443	0.001628	-0.000419
$ErCl_3$	0.259748	-0.009487	-0.003585	-0.004234	0.000984

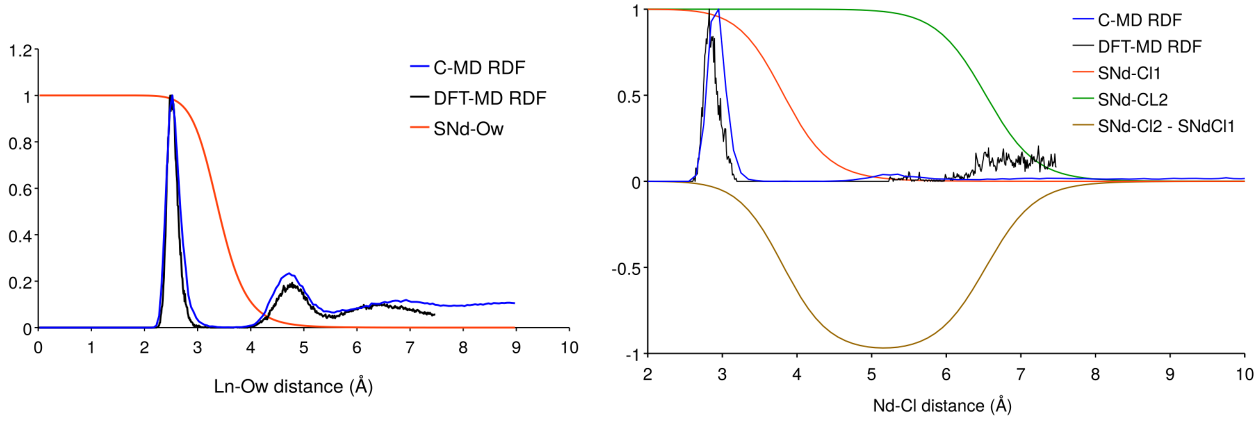

Figure S1 *Normalised functions, S, used to define Nd-Ow coordination (left) and Nd-Cl coordination in the first,* $S_{Nd\text{-}Cl1}$*, and within the first two,* $S_{Nd\text{-}Cl2}$*, coordination spheres (right). Also plotted are normalised RDFs for the atom pairs given in axis labels. Both DFT-MD and C-MD data are provided for comparison. Right also shows* $S_{Nd\text{-}Cl1}$ *-* $S_{Nd\text{-}Cl2}$*, which highlights the regions where there is minimal overlap between the two coordination functions.*

Table S2 *Parameters used to define continuous functions, S, for Ln coordination. See main text, Equation 4 for the function definition. Label subscripts show atom types, with Cl1 and Cl2 referring to the first and first two Ln shell coordinated Cl ions, respectively. Parameters were chosen such that the functions go smoothly from one to zero between the first and second or third peaks in the relevant normalised RDFs (see Figure 1 above); furthermore, the parameters ensure that the values at which S and normalised RDFs cross-over were equal between different* $LnCl_3$*(aq) systems and, hence, direct comparison between coordination levels in all systems can be made.*

	d_0	r_0	n	m
$S_{Nd\text{-}Ow}$	0.8	2.6	10	20
$S_{Nd\text{-}Cl1}$	0.85	3.1	8	20
$S_{Nd\text{-}Cl2}$	1	5.65	14	34
$S_{Gd\text{-}Ow}$	0.8	2.375	10	19
$S_{Gd\text{-}Cl1}$	0.82	2.85	8	18
$S_{Gd\text{-}Cl2}$	1	5.65	14	34
$S_{Er\text{-}Ow}$	0.8	2.325	10	19
$S_{Er\text{-}Cl1}$	0.8	2.75	10	18
$S_{Er\text{-}Cl2}$	1	5.4	14	34

[1] Spedding et al., "Densities and Apparent Molal Volumes of Some Aqueous Rare Earth Solutions at 25.Deg. I. Rare Earth Chlorides."

Table S3 *CV (collective variable) values and restraint constants, k, in Nd-Cl 2D Umbrella Sampling calculations. CV1 is a distance parameter in Angstrom units, CV2 is a coordination number and k are provided in kJ/mol.*

CV1=2.50, k=100.0; CV2=6.5, k=125.0
CV1=2.50, k=100.0; CV2=7.0, k=50.0
CV1=2.50, k=100.0; CV2=7.5, k=50.0
CV1=2.50, k=100.0; CV2=8.0, k=50.0
CV1=2.50, k=100.0; CV2=8.5, k=50.0
CV1=2.50, k=100.0; CV2=9.0, k=50.0
CV1=2.50, k=200.0; CV2=6.0, k=125.0
CV1=2.50, k=200.0; CV2=6.5, k=125.0
CV1=2.50, k=200.0; CV2=7.0, k=50.0
CV1=2.50, k=200.0; CV2=7.5, k=50.0
CV1=2.50, k=200.0; CV2=8.0, k=50.0
CV1=2.50, k=200.0; CV2=8.5, k=50.0
CV1=2.50, k=200.0; CV2=9.0, k=50.0
CV1=2.75, k=100.0; CV2=6.5, k=125.0
CV1=2.75, k=100.0; CV2=7.0, k=50.0
CV1=2.75, k=100.0; CV2=7.5, k=50.0
CV1=2.75, k=100.0; CV2=8.0, k=50.0
CV1=2.75, k=100.0; CV2=8.5, k=50.0
CV1=2.75, k=100.0; CV2=9.0, k=50.0
CV1=3.00, k=50.0; CV2=6.5, k=125.0
CV1=3.00, k=50.0; CV2=7.0, k=50.0
CV1=3.00, k=50.0; CV2=7.5, k=50.0
CV1=3.00, k=50.0; CV2=8.0, k=50.0
CV1=3.00, k=50.0; CV2=8.5, k=50.0
CV1=3.00, k=50.0; CV2=9.0, k=50.0
CV1=3.25, k=100.0; CV2=7.0, k=50.0
CV1=3.25, k=100.0; CV2=7.5, k=50.0
CV1=3.25, k=100.0; CV2=8.0, k=50.0
CV1=3.25, k=100.0; CV2=8.5, k=50.0
CV1=3.25, k=100.0; CV2=9.0, k=50.0
CV1=3.50, k=150.0; CV2=7.0, k=50.0
CV1=3.50, k=150.0; CV2=7.5, k=50.0
CV1=3.50, k=150.0; CV2=8.0, k=50.0
CV1=3.50, k=150.0; CV2=8.5, k=50.0
CV1=3.50, k=150.0; CV2=9.0, k=50.0
CV1=3.75, k=150.0; CV2=7.0, k=50.0
CV1=3.75, k=150.0; CV2=7.5, k=50.0
CV1=3.75, k=150.0; CV2=8.0, k=50.0
CV1=3.75, k=150.0; CV2=8.5, k=125.0
CV1=3.75, k=150.0; CV2=9.0, k=50.0
CV1=3.75, k=150.0; CV2=9.5, k=125.0
CV1=4.00, k=150.0; CV2=7.0, k=50.0
CV1=4.00, k=150.0; CV2=7.5, k=50.0
CV1=4.00, k=150.0; CV2=8.0, k=50.0
CV1=4.00, k=150.0; CV2=8.5, k=125.0
CV1=4.00, k=150.0; CV2=9.0, k=50.0
CV1=4.00, k=150.0; CV2=9.5, k=125.0
CV1=4.25, k=150.0; CV2=7.0, k=50.0
CV1=4.25, k=150.0; CV2=7.5, k=50.0
CV1=4.25, k=150.0; CV2=8.0, k=50.0
CV1=4.25, k=150.0; CV2=8.5, k=125.0
CV1=4.25, k=150.0; CV2=9.0, k=50.0
CV1=4.25, k=150.0; CV2=9.5, k=125.0
CV1=4.50, k=150.0; CV2=7.0, k=50.0
CV1=4.75, k=100.0; CV2=8.5, k=125.0
CV1=4.75, k=100.0; CV2=9.0, k=50.0
CV1=4.75, k=100.0; CV2=9.5, k=125.0
CV1=5.00, k=50.0; CV2=7.0, k=50.0
CV1=5.00, k=50.0; CV2=7.5, k=50.0
CV1=5.00, k=50.0; CV2=8.0, k=50.0
CV1=5.00, k=50.0; CV2=8.5, k=125.0
CV1=5.00, k=50.0; CV2=9.0, k=50.0
CV1=5.00, k=50.0; CV2=9.5, k=125.0
CV1=5.50, k=50.0; CV2=7.0, k=50.0
CV1=5.50, k=50.0; CV2=7.5, k=50.0
CV1=5.50, k=50.0; CV2=8.0, k=50.0
CV1=5.50, k=50.0; CV2=8.5, k=125.0
CV1=5.50, k=50.0; CV2=9.0, k=50.0
CV1=5.50, k=50.0; CV2=9.5, k=125.0
CV1=6.00, k=50.0; CV2=7.0, k=50.0
CV1=6.00, k=50.0; CV2=7.5, k=50.0
CV1=6.00, k=50.0; CV2=8.0, k=50.0
CV1=6.00, k=50.0; CV2=8.5, k=125.0
CV1=6.00, k=50.0; CV2=9.0, k=50.0
CV1=6.00, k=50.0; CV2=9.5, k=125.0
CV1=6.50, k=50.0; CV2=7.0, k=50.0
CV1=6.50, k=50.0; CV2=7.5, k=50.0
CV1=6.50, k=50.0; CV2=8.0, k=50.0
CV1=6.50, k=50.0; CV2=8.5, k=125.0
CV1=6.50, k=50.0; CV2=9.0, k=50.0
CV1=6.50, k=50.0; CV2=9.5, k=125.0
CV1=7.00, k=50.0; CV2=7.0, k=50.0
CV1=7.00, k=50.0; CV2=7.5, k=50.0
CV1=7.00, k=50.0; CV2=8.0, k=50.0
CV1=7.00, k=50.0; CV2=8.5, k=125.0
CV1=7.00, k=50.0; CV2=9.0, k=50.0
CV1=7.00, k=50.0; CV2=9.5, k=125.0
CV1=7.50, k=50.0; CV2=7.0, k=50.0
CV1=7.50, k=50.0; CV2=7.5, k=50.0
CV1=7.50, k=50.0; CV2=8.0, k=50.0
CV1=7.50, k=50.0; CV2=8.5, k=125.0
CV1=7.50, k=50.0; CV2=9.0, k=50.0
CV1=7.50, k=50.0; CV2=9.5, k=125.0
CV1=8.00, k=50.0; CV2=7.0, k=50.0
CV1=8.00, k=50.0; CV2=7.5, k=50.0
CV1=8.00, k=50.0; CV2=8.0, k=50.0
CV1=8.00, k=50.0; CV2=8.5, k=125.0
CV1=8.00, k=50.0; CV2=9.0, k=50.0
CV1=8.00, k=50.0; CV2=9.5, k=125.0
CV1=8.50, k=50.0; CV2=7.0, k=50.0
CV1=8.50, k=50.0; CV2=7.5, k=50.0
CV1=8.50, k=50.0; CV2=8.0, k=50.0
CV1=8.50, k=50.0; CV2=8.5, k=125.0
CV1=8.50, k=50.0; CV2=9.0, k=50.0
CV1=8.50, k=50.0; CV2=9.5, k=125.0
CV1=9.00, k=50.0; CV2=7.0, k=50.0
CV1=9.00, k=50.0; CV2=7.5, k=50.0
CV1=9.00, k=50.0; CV2=8.0, k=50.0

CV1=4.50, k=150.0; CV2=7.5, k=50.0
CV1=4.50, k=150.0; CV2=8.0, k=50.0
CV1=4.50, k=150.0; CV2=8.5, k=125.0
CV1=4.50, k=150.0; CV2=9.0, k=50.0
CV1=4.50, k=150.0; CV2=9.5, k=125.0
CV1=4.75, k=100.0; CV2=7.0, k=50.0
CV1=4.75, k=100.0; CV2=7.5, k=50.0
CV1=4.75, k=100.0; CV2=8.0, k=50.0
CV1=9.50, k=50.0; CV2=9.5, k=125.0
CV1=10.0, k=50.0; CV2=7.0, k=50.0
CV1=10.0, k=50.0; CV2=7.5, k=50.0
CV1=10.0, k=50.0; CV2=8.0, k=50.0
CV1=10.0, k=50.0; CV2=8.5, k=125.0
CV1=10.0, k=50.0; CV2=9.0, k=50.0
CV1=10.0, k=50.0; CV2=9.5, k=125.0
CV1=10.5, k=50.0; CV2=7.0, k=50.0
CV1=10.5, k=50.0; CV2=7.5, k=50.0
CV1=10.5, k=50.0; CV2=8.0, k=50.0
CV1=10.5, k=50.0; CV2=8.5, k=125.0
CV1=10.5, k=50.0; CV2=9.0, k=50.0
CV1=10.5, k=50.0; CV2=9.5, k=125.0
CV1=11.0, k=50.0; CV2=7.0, k=50.0
CV1=11.0, k=50.0; CV2=7.5, k=50.0
CV1=11.0, k=50.0; CV2=8.0, k=50.0
CV1=11.0, k=50.0; CV2=8.5, k=125.0
CV1=11.0, k=50.0; CV2=9.0, k=50.0
CV1=11.0, k=50.0; CV2=9.5, k=125.0
CV1=11.5, k=50.0; CV2=7.0, k=50.0
CV1=11.5, k=50.0; CV2=7.5, k=50.0
CV1=11.5, k=50.0; CV2=8.0, k=50.0
CV1=11.5, k=50.0; CV2=8.5, k=125.0
CV1=11.5, k=50.0; CV2=9.0, k=50.0
CV1=11.5, k=50.0; CV2=9.5, k=125.0
CV1=12.0, k=50.0; CV2=7.0, k=50.0
CV1=12.0, k=50.0; CV2=7.5, k=50.0
CV1=12.0, k=50.0; CV2=8.0, k=50.0
CV1=12.0, k=50.0; CV2=8.5, k=125.0
CV1=12.0, k=50.0; CV2=9.0, k=50.0
CV1=12.0, k=50.0; CV2=9.5, k=125.0
CV1=12.5, k=50.0; CV2=7.0, k=50.0
CV1=12.5, k=50.0; CV2=7.5, k=50.0
CV1=12.5, k=50.0; CV2=8.0, k=50.0
CV1=12.5, k=50.0; CV2=8.5, k=125.0
CV1=12.5, k=50.0; CV2=9.0, k=50.0
CV1=12.5, k=50.0; CV2=9.5, k=125.0
CV1=9.00, k=50.0; CV2=8.5, k=125.0
CV1=9.00, k=50.0; CV2=9.0, k=50.0
CV1=9.00, k=50.0; CV2=9.5, k=125.0
CV1=9.50, k=50.0; CV2=7.0, k=50.0
CV1=9.50, k=50.0; CV2=7.5, k=50.0
CV1=9.50, k=50.0; CV2=8.0, k=50.0
CV1=9.50, k=50.0; CV2=8.5, k=125.0
CV1=9.50, k=50.0; CV2=9.0, k=50.0
CV1=13.0, k=50.0; CV2=7.0, k=50.0
CV1=13.0, k=50.0; CV2=7.5, k=50.0
CV1=13.0, k=50.0; CV2=8.0, k=50.0
CV1=13.0, k=50.0; CV2=8.5, k=125.0
CV1=13.0, k=50.0; CV2=9.0, k=50.0
CV1=13.0, k=50.0; CV2=9.5, k=125.0
CV1=13.5, k=50.0; CV2=7.0, k=50.0
CV1=13.5, k=50.0; CV2=7.5, k=50.0
CV1=13.5, k=50.0; CV2=8.0, k=50.0
CV1=13.5, k=50.0; CV2=8.5, k=125.0
CV1=13.5, k=50.0; CV2=9.0, k=50.0
CV1=13.5, k=50.0; CV2=9.5, k=125.0
CV1=14.0, k=50.0; CV2=7.0, k=50.0
CV1=14.0, k=50.0; CV2=7.5, k=50.0
CV1=14.0, k=50.0; CV2=8.0, k=50.0
CV1=14.0, k=50.0; CV2=8.5, k=125.0
CV1=14.0, k=50.0; CV2=9.0, k=50.0
CV1=14.0, k=50.0; CV2=9.5, k=125.0
CV1=14.5, k=50.0; CV2=7.0, k=50.0
CV1=14.5, k=50.0; CV2=7.5, k=50.0
CV1=14.5, k=50.0; CV2=8.0, k=50.0
CV1=14.5, k=50.0; CV2=8.5, k=125.0
CV1=14.5, k=50.0; CV2=9.0, k=50.0
CV1=14.5, k=50.0; CV2=9.5, k=125.0
CV1=15.0, k=50.0; CV2=7.0, k=50.0
CV1=15.0, k=50.0; CV2=7.5, k=50.0
CV1=15.0, k=50.0; CV2=8.0, k=50.0
CV1=15.0, k=50.0; CV2=8.5, k=125.0
CV1=15.0, k=50.0; CV2=9.0, k=50.0
CV1=15.0, k=50.0; CV2=9.5, k=125.0
CV1=15.5, k=50.0; CV2=7.0, k=50.0
CV1=15.5, k=50.0; CV2=7.5, k=50.0
CV1=15.5, k=50.0; CV2=8.0, k=50.0
CV1=15.5, k=50.0; CV2=8.5, k=125.0
CV1=15.5, k=50.0; CV2=9.0, k=50.0
CV1=15.5, k=50.0; CV2=9.5, k=125.0

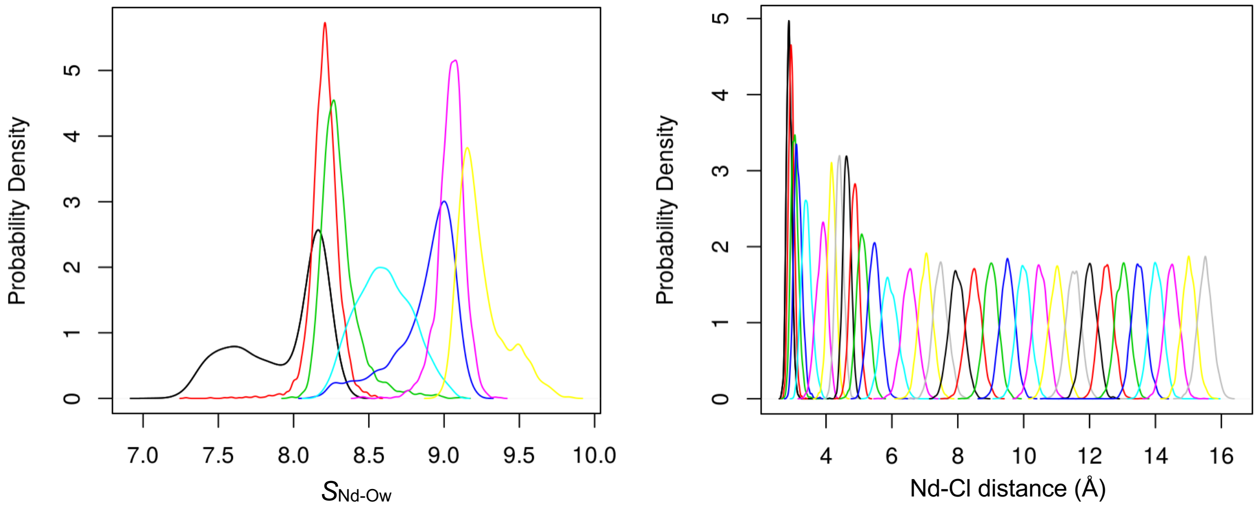

Figure S2 *Probability density distributions for Umbrella Sampling windows in Nd-Cl calculations. Left shows water coordination around Nd^{3+}, as defined by the continuous function $S_{Nd\text{-}Ow}$ (see main text Equation 4), while right shows Nd-Cl distances. Colours highlight adjacent windows.*

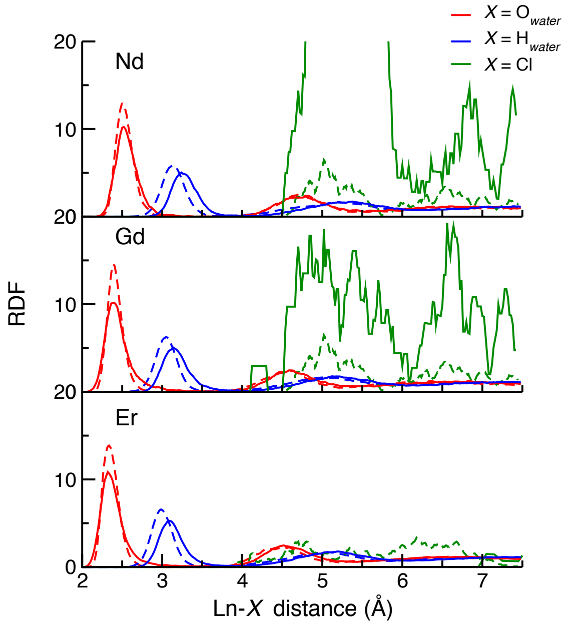

Figure S3 *Radial distribution functions (RDFs) calculated from time averaged simulations of $LnCl_3$ in water. RDFs from C-MD are shown by solid lines and from DFT-MD by dashed lines.*

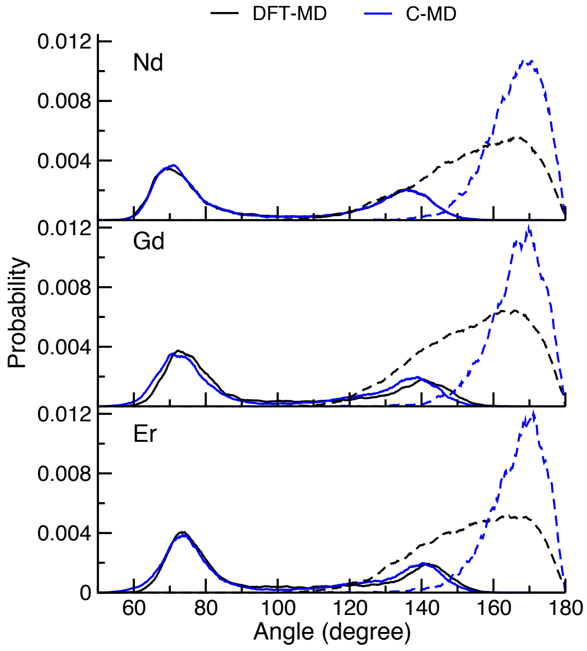

Figure S4 *Ln(III)-water angle probability distribution for inner sphere water molecules. The probabilities for O–Ln–O angles and Ln–O–M angles in the first coordination sphere of Ln(III) are given by solid and dashed lines, respectively. M represents the bisector formed between the two O–H bonds of a water molecule and so this angle represents the tilt of water molecules surrounding the cation. DFT-MD and C-MD from 12 ps and 2.5 ns trajectories are shown, respectively.*

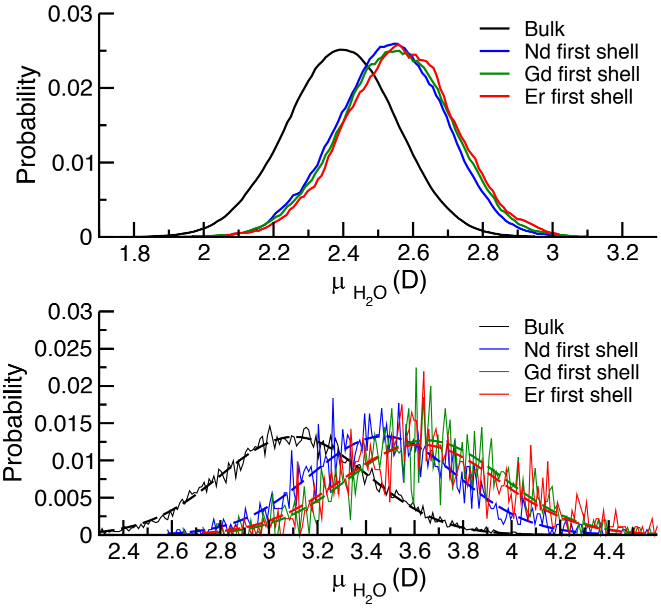

Figure S5 *Water dipole moments, μ_{H_2O}, in Ln(III) solutions. Dipole moment probability distributions, $P(\mu_{H_2O})$, for water in the first Ln(III) coordination sphere are given as well as those for bulk water (defined as water molecules outside of the second Ln(III) shell). Top shows C–MD distributions from 2.5 ns trajectories, while those from 12 ps DFT–MD calculations are bottom. In the case of DFT–MD calculations, the solid and dashed lines show the calculated and fitted distributions, respectively. The fits were performed using Gaussian functions:* $P(\mu_{H_2O}) = A_0 \exp\left(\frac{\mu_{H_2O}-A_1}{2A_2^2}\right)$ *where A_1 are the positions of the peak centres and A_2 are associated standard deviations. Values of A_0= 0.013, 0.013, 0.013, 0.012, A_1= 3.10, 3.47, 3.64, 3.62 and A_2= 0.30, 0.30, 0.32, 0.32 were determined for bulk water and Nd, Gd and Er first shell water molecules, respectively.*

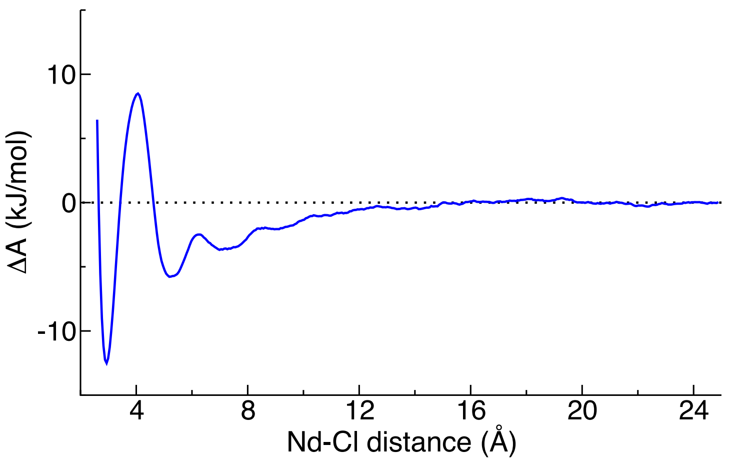

Figure S6 *Relative free energy for Nd-Cl binding as a function of cation-anion distance as calculated in one-dimensional Umbrella Sampling calculations as a function of Nd-Cl separation distance. 50 US windows were simulated in the distance range shown in the plot in 0.5 Å increments of the collective variable. An entropy correction, given in main text Equation 6 and in the last section of this SI, was applied. The curve has been shifted to the zero line for dissociated ions at large distances.*

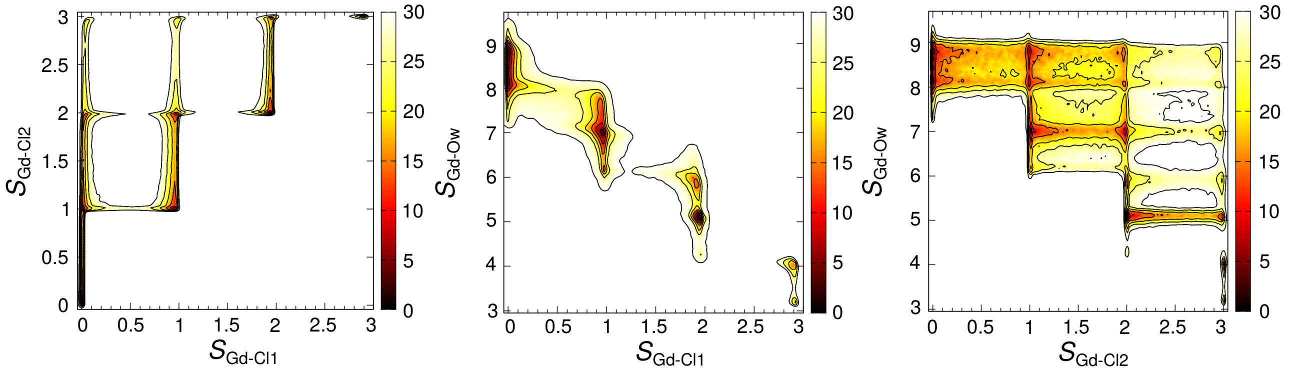

Figure S7 *Potential of mean force energies for Gd speciation by Cl in aqueous solution. Relative energies (in kJ mol^{-1} units) are shown by the colour scale on the right. Metadynamics calculations were performed which involved the time dependent biased sampling of Gd with oxygen of water (S_{Gd-O}) and chloride (S_{Gd-Cl1}) in the first coordination sphere and chloride coordination within the first two spheres (S_{Gd-Cl2}). Plots show projections of the four-dimensional energy surface onto two-dimensional reaction coordinates. Contour lines indicate energies of $2k_BTN_A$.*

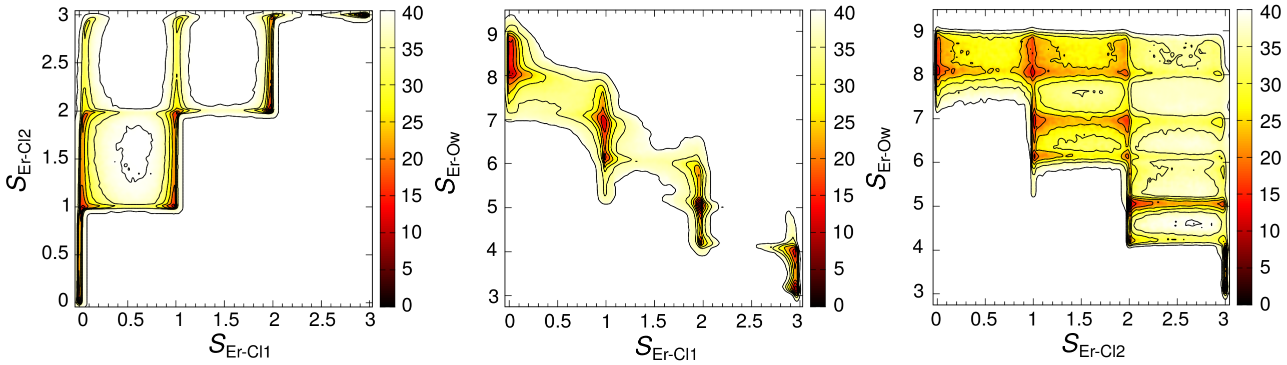

Figure S8 *Potential of mean force energies for Er speciation by Cl in aqueous solution. Relative energies (in kJ mol^{-1} units) are shown by the colour scale on the right. Metadynamics calculations were performed which involved the time dependent biased sampling of Er with oxygen of water ($S_{\mathrm{Er-O}}$) and chloride ($S_{\mathrm{Er-Cl1}}$) in the first coordination sphere and chloride coordination within the first two spheres ($S_{\mathrm{Er-Cl2}}$). Plots show projections of the four-dimensional energy surface onto two-dimensional reaction coordinates. Contour lines indicate energies of 2 $k_B N_A T$.*

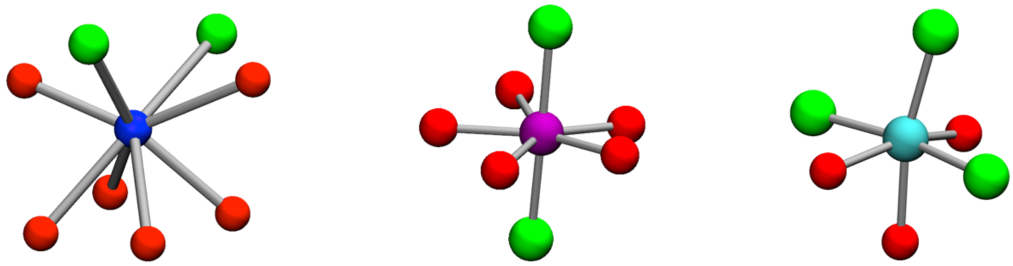

Figure S9 *Snapshots of the configurations for [$NdCl_2(OH_2)_6$]$^+$ (left), [$GdCl_2(OH_2)_5$]$^+$ (middle) and [$ErCl_3(OH_2)_3$]^0 (right) as observed in metadynamics calculations. Blue, purple, cyan, green and red spheres show Nd, Gd, Er, Cl and O atoms, respectively. Lines highlight coordination between ligand atoms and Nd.*

Calculating Equilibrium Constants

As discussed in the main text, it is possible to calculate stability constants, β, from the two-dimensional (Ln–Cl distance (s_1) and Ln-Ow coordination (s_2)) potential of mean force ($\mathcal{W}$) energy landscapes shown in main text Figure 4. First, a dimensionality reduction is performed to examine $\mathcal{W}$ as a function of just Ln–Cl distance:

$$\mathcal{W}(s_1) = -k_{\mathrm{B}} N_{\mathrm{A}} T \ln \int \exp\left(\frac{-\mathcal{W}(s_1, s_2)}{k_{\mathrm{B}} N_{\mathrm{A}} T}\right) ds_2 \qquad \text{Eq. 1}$$

where k_{B}, N_{A} and T are the Boltzmann constant, Avogadro's constant and temperature, respectively. A correction must then be added to account for the diffusional driving force to increase ion separation, resulting in a favourable increase in entropy, given that s_1 is a function of the distance r. This results in the relative free energy, ΔA,

$$\Delta A(r) = \mathcal{W}(r) + 2k_{\mathrm{B}} N_{\mathrm{A}} T\,[\ln(r) - \ln(r_c)] \qquad \text{Eq. 2}$$

where r_c is some reference distance which, in our case, was the maximum distance in the free energy profiles which result from Equation 1.

The free energy profiles for the three cation-anion reactions studied are provided in main text Figure 5. From these it is possible to calculate equilibrium constants. For a full derivation, please see the paper by Chialvo *et al.*[2], otherwise the main formulae used in this work will be provided below.

[2] A. A. Chialvo *et al.*, *J. Chem. Phys.*, **1995**, *103(21)*, 9379-9387

The association equilibrium constant, K_a, for forming ion pairs (ip) can be written as,

$$K_a = \frac{\gamma_{ip}\ c(\mathrm{LnCl}^{2+})}{\gamma_+\ c(\mathrm{Ln}^{3+})\ \gamma_-\ c(\mathrm{Cl}^-)} = \frac{\gamma_{ip}\ \rho_{ip}}{\gamma_+\ \rho_{Ln}\ \gamma_-\ \rho_{Cl}} \qquad \text{Eq. 3}$$

where γ, c and ρ refer to the species activity coefficients (subscript $+$ and $-$ refer to cations and anions), concentrations and number densities ($\rho = N/V$ where N is the number of ions/ion pairs and V is the simulation cell volume) of species. Given a degree of association between a cations and anions, α, Equation 3 can be rewritten as follows,

$$K_a = \frac{\gamma_{ip}\ \alpha}{\gamma_+\ \gamma_-\ \rho_0\ (1-\alpha)^2} \qquad \text{Eq. 4}$$

This equation assumes that the cation binds to a single anion. ρ_0 is the total number density of ion pairs which in our case is $1/V$. The number densities of ions can be calculated from radial distribution functions, which in turn can be calculated via integration of the Boltzmann weighted free energies given by Equation 2:

$$K_a = \frac{\gamma_{ip} \int_{r_0}^{r_m} dr\ \ 4\pi r^2 \exp\left(\frac{-\Delta A}{k_\mathrm{B} N_\mathrm{A} T}\right)}{\gamma_+\ \gamma_-\ \rho_0 \left(1 - \int_{r_0}^{r_m} dr\ \ 4\pi r^2 \exp\left(\frac{-\Delta A}{k_\mathrm{B} N_\mathrm{A} T}\right)\right)^2} \qquad \text{Eq. 5}$$

The choice of r_0 and r_m can significantly affect the value of the association constant that is calculated. These limits may differ depending upon how an ion pair is defined. In our case, r_0 was the minimum value in the free energy profiles and r_m was 15 Å, justification for which is provided in the main text.

As discussed in the main text, it is often useful to assume that ions are at infinite dilution which is not unreasonable given the nature of the calculation performed. In this case, the activity coefficients are unity and we can write the association constant as,

$$\lim_{\rho \to 0} K_a = \int_{r_0}^{r_m} dr\ 4\pi r^2 \exp\left(\frac{-\Delta A}{k_\mathrm{B} N_\mathrm{A} T}\right) \qquad \text{Eq. 6}$$

Returning to Equation 5, the only unknowns are the activity coefficients for the ion pair and free ions. In experiments, the Debye-Hückel theory is used to calculate activity coefficients. Given the molalities, b, for cations in this work ($b(\mathrm{Ln}^{3+})$ = 0.01 mol kg^{-1}, $b(\mathrm{Cl}^-)$ = 0.03 mol kg^{-1}) we opted for an extension of the Debye-Hückel limiting law:

$$-\log_{10}(\gamma) = \frac{A|z|\sqrt{I}}{1 + Ba\sqrt{I}} \qquad \text{Eq. 7}$$

where z is the value of electronic charge on ions in units of e. A and B are temperature and dielectric dependent constants which are 0.5085 and 0.3281 mol$^{1/2}$kg$^{1/2}$ for water at 298 K.[3] Ionic strength, I, was calculated according to the total molalities of ions in the simulations,

$$I = \frac{1}{2}\left(z_{Ln}^2 b(\mathrm{Ln}) + z_{Cl}^2 b(\mathrm{Cl})\right) \qquad \text{Eq. 8}$$

For the simulations in this work, the ionic strength was 0.06 mol/kg.

a in Equation 7 is the mean diameter of ions, providing a distance of closest approach. Measuring a by experiment can be challenging.[4] For example, a number of studies in the literature (and cited in the current work) take mean a values from a seminal paper by Kielland[5] but the variations in individual measurements contributing to these mean values are large. Simulations can play an important role here because the pair correlation function of ions and water can be computed easily by time averaging sampled microstates.

The ion–O_{water} RDFs were calculated from 2 ns simulations of single ions in water at 0.01 mol kg^{-1} with all other simulation parameters as described in the methods section of the main paper. A box of 5550 SPC/Fw molecules was also simulated for 2 ns to calculate O_{water}–O_{water} RDFs. In addition, the minima in the free energy profiles in Figure 5 of the main paper provide the most probable cation-anion separation distances for CIPs and SShIPs.

---

[3] G. G. Manov *et al.*, *J. Am. Chem. Soc.*, **1943**, *65* (9), 1765–1767.
[4] Y. Marcus, *Chem. Rev.*, **1988**, 88, 1475–1498.
[5] J. Kielland, *J. Am. Chem. Soc.*, **1937**, *59* (9), 1675–1678.

The radius of a water molecule, R_{water}, was defined by half of the distance measured at the position of the first maximum in O_{water}–O_{water} RDFs from a simulation of pure water. Marcus[3] defines the ion radii in solution as,

$$R_{ion} = d^{(1)}_{ion-O} - R_{water} \quad \text{Eq. 9}$$

where $d^{(1)}_{ion-O}$ is the position of the first maximum in the ion–O_{water} RDFs. R_{ion}values by this definition were 1.159, 1.022 and 0.956 Å for Nd^{3+}, Gd^{3+} and Er^{3+}, respectively, and in good agreement with the mean computed values provided by Marcus[3].

The radii of ion pairs was calculated as,

$$R_{CIP} = \frac{a_{CIP}}{2} = s^{(1)}_{A} + R_{Ln} + R_{Cl}$$
$$R_{SShIP} = \frac{a_{SShIP}}{2} = s^{(2)}_{A} + R_{Ln} + R_{Cl} \quad \text{Eq. 10}$$

where $s^{(1)}_{A}$ and $s^{(2)}_{A}$ were the positions of the first and second minima, respectively, in the free energy profiles in main text Figure 5. Table 4 provides the a values that were used to compute Equation 7.

Table 4 *Ion diameters, a, used in this work.*

	a (Å)
O_{water}	2.76
Cl^{-}	3.9
Nd^{3+}	2.32
Gd^{3+}	2.04
Er^{3+}	1.91
CIP–$[NdCl]^{2+}$	6.01
CIP–$[GdCl]^{2+}$	5.71
CIP–$[ErCl]^{2+}$	5.55
SShIP–$[NdCl]^{2+}$	8.29
SShIP–$[GdCl]^{2+}$	8.14
SShIP–$[ErCl]^{2+}$	8

Using Equation 5, we calculated K_a to be 1.677, 1.595 and 1.582 for Nd^{3+}, Gd^{3+} and Er^{3+}, respectively, in units of $dm^3\ mol^{-1}$. These are consistently larger than the values when infinite dilution is assumed (as reported in the main text as 1.259, 1.198 and 1.88 $dm^3\ mol^{-1}$ for Nd^{3+}, Gd^{3+} and Er^{3+}, respectively). This is intuitively sensible—moving from an ideal solution regime to a non-ideal one should result in increased association due to the favourable interactions between cations and anions. Both approaches to estimate K_a give values which deviate from the experimental predictions. However, the trend to decreasing values is evident in both and $\Delta \log(K_a)\ /\Delta Z$ (where Z is atomic number) is-0.01 independent on the approach taken.
